# Validation of cognitive and psychosocial tools in Kenya: findings from the LOSHAK feasibility pilot

**DOI:** 10.1186/s12889-025-24918-z

**Published:** 2025-11-17

**Authors:** Sneha Sarah Mani, Roselyter Monchari Riang’a, Niranjani Nagarajan, Anthony K. Ngugi, Eunice Muthoni Mwangi, Muthoni Gichu, Patrick N. Mwangala, Edward Miguel, Michael Walker, Kenneth M. Langa, Joshua R. Ehrlich, Alden L. Gross

**Affiliations:** 1 Department of Epidemiology, School of Public Health, Johns Hopkins University, Baltimore, USA; 2 Department of Population Health, Medical College, Aga Khan University, Nairobi, Kenya; 3 Department of Ophthalmology and Visual Sciences, University of Michigan, Ann Arbor, USA; 4 Division of Palliative and Aged Care, Ministry of Health, Nairobi, Kenya; 5 Institute for Human Development, Aga Khan University, Nairobi, Kenya; 6 University of California - Berkeley, Berkeley, USA; 7 Survey Research Center, Institute for Social Research, University of Michigan, Ann Arbor, USA; 8 Internal Medicine, University of Michigan, Ann Arbor, USA; 9 Ann Arbor Veterans Affairs Hospital, Ann Arbor, MI USA

**Keywords:** Cognition, Dementia, Successful aging, Sub-Saharan Africa, Validation

## Abstract

**Background and objectives:**

Adapting cognitive tests to culturally diverse and low-resource settings is essential for expanding knowledge of cognitive health in older adults beyond high-income countries. However, contextual differences in novel settings can affect reliability and validity. There is limited evidence on the feasibility of implementing culturally adapted cognitive assessments in Sub-Saharan Africa, a region projected to have over 158 million adults aged 60 and older by 2050. The Longitudinal Study of Health and Ageing in Kenya (LOSHAK) is designed to address this measurement gap by evaluating cognitive tests in this context.

**Research design and methods:**

We analyzed data from 205 adults aged 45 years and older from the Kaloleni/Rabai Health and Demographic Surveillance System in Coastal Kenya. Using McDonald’s omega and confirmatory factor analysis, we evaluated the internal consistency reliability and the factor structure of four cognitive domains (orientation, memory, executive functioning, language/fluency) and four psychosocial constructs (depressive symptoms, loneliness, subjective well-being, and life satisfaction).

**Results:**

McDonald’s omegas ranged from ω = 0.78 to 0.95 for cognitive domains and were above 0.83 for each psychosocial domain, suggesting high reliability. Factor analyses revealed adequate to perfect fit for most domains, and patterns of factor loadings were mostly acceptable.

**Conclusion:**

The successful adaptation of these assessment tools in Kenya demonstrates the feasibility of implementing rigorous cognitive and psychosocial measurements in low-resource settings. These findings provide a methodological framework for future aging studies in similar contexts across Sub-Saharan Africa.

**Supplementary Information:**

The online version contains supplementary material available at 10.1186/s12889-025-24918-z.

## Background

Adapting cognitive tests to culturally diverse, particularly low-resource settings, is essential for expanding knowledge of cognitive health in older adults beyond high-income countries [1]. Contextual differences can affect the reliability and validity of existing neuropsychological and psychosocial assessments [2–4]. The absence of rigorous studies evaluating the validity of existing cognitive assessment tools in low- and middle-income regions has contributed to a considerable knowledge gap in our understanding of cognitive health among older adults in these regions [4, 5]. While there has been progress with recent studies in South Africa, China, India, Mexico, and Chile that have implemented the Harmonized Cognitive Assessment Protocol (HCAP) [6, 7], studies from the Sub-Saharan region in Africa have been limited [8]. To understand the factors associated with Alzheimer’s disease and related dementias (AD/ADRD) and to reduce the attributable global burden of disease, cross-national comparisons using high-quality data based on harmonized instruments are essential [5]. This is especially important in Sub-Saharan Africa (SSA) since approximately 75% of ADRD cases by 2050 are projected to occur in low- and middle-income countries (LMICs) [9]. 

SSA is expected to be home to over 158 million older adults by 2050 [10]. Furthermore, by 2100, the average annual growth rate of the population aged 60 and above in SSA is projected to surpass the total population growth rate of other world regions, as well as the older population growth rates in populous countries such as India or China [11, 12]. In recent years, health research and investments in SSA have focused on nutritional, communicable, and infectious diseases affecting children, mothers, and working-age adults [13]. However, the SSA region must now also address the growing prevalence of non-communicable diseases, including dementia, in the upcoming decades, especially among older adults [13–15]. To date, the only dementia prevalence estimate in SSA using a harmonized cognitive assessment protocol (HCAP) comes from the Health and Aging in Africa: A Longitudinal Study in South Africa (HAALSI), which reported a dementia prevalence of 18% in the Agincourt community [16]. This estimate was derived from cognitive testing, informant interviews, and neurological assessments. To better characterize the burden of dementia in the region, additional population-based studies incorporating repeated measures of cognitive function and daily functioning are urgently needed from other regions in SSA [16]. 

Longitudinal Study of Health and Aging in Kenya (LOSHAK), the second study in the Health and Retirement Study’s network of harmonized studies on aging in SSA, aims to build this infrastructure and test data collection protocols for a population-based panel study on health and aging in Kenya [8]. The study will address critical data gaps and contribute to the global network of aging studies, allowing for cross-national comparisons [8]. While earlier studies such as the Health and Aging in Africa: A Longitudinal Study of an INDEPTH Community in South Africa (HAALSI) and the WHO Study on Global AGEing and Adult Health (SAGE) in Ghana have provided valuable insights into aging in the region, LOSHAK expands this work by targeting a more diverse Kenyan population aged 45 and older [8]. While Alzheimer’s disease typically affects individuals older than 65, changes in the brain can begin years before [17]. Including middle-aged adults in the LOSHAK sample provides a valuable opportunity to observe individuals before the onset of cognitive decline and to track cognitive changes as they age. Future waves of data collection will also capture additional health conditions like cardiovascular diseases [8] associated with cognitive outcomes, further enriching the study of cognitive aging.

LOSHAK completed a feasibility and pilot study of health among adults in the Kaloleni/Rabai Health and Demographic Surveillance System, aiming to address measurement and knowledge gaps related to cognitive testing in 2023. In the current pilot, we administered neuropsychological and psychosocial assessments drawn from the Health and Retirement Study (HRS) and Harmonized Cognitive Assess Protocol (HCAP) families of studies, adapted to the Kenyan context [8]. We evaluated psychometric characteristics of neuropsychological and psychosocial assessments administered as part of the feasibility and pilot phase of LOSHAK, to improve our understanding of the applicability and limitations when adapting existing instruments in this context.

## Methods

### Study design and participants

The feasibility and pilot study participants were drawn from the Kaloleni/Rabai Health and Demographic Surveillance System (KRHDSS) [8] in Coastal Kenya [18], and the study was conducted between March and May 2023. The final pilot sample of 205, identified through sampling proportionate to the distribution of population by age from the KRHDSS participants, includes 41% aged 45–55 years, 31% aged 56–65 years, and 28% over 65 years, and are approximately equally distributed by gender and location of residence (rural and peri-urban). The LOSHAK survey instrument (see Additional File 1) included assessments of cognitive performance and psychosocial tests, as well as mental health, behavioral and other self-reported health measures, caregiving information, demographic information of the household and respondent, measures of environmental exposures, income and assets, economics, and physiological measures.

Multiple initiatives were undertaken to ensure the study used culturally responsive data collection approaches, including recruiting local enumerators, framing study goals within local values, customs, and traditions, and engaging local stakeholders. These strategies are described in detail elsewhere [19]. The Institutional Scientific and Ethical Review Committee at Aga Khan University, Kenya, and the Institutional Review Board at the University of Michigan reviewed and approved the LOSHAK study protocol and survey instruments. All participants provided informed consent. If participants were unable to consent due to cognitive impairment, a close informant provided consent. For those who were illiterate or unable to sign, consent was documented via a witnessed thumb impression after the form was read aloud. All consent forms and surveys were translated into Swahili and back-translated according to WHO standards to ensure accuracy [8]. 

### Variables

Cognitive and psychosocial instruments in LOSHAK were adapted from the US HRS and partner studies to be relevant in the Kenyan context (see Additional File 1). For example, the tests included a 21-item Swahili Mental State Examination, which was adapted from the Hindi Mental State Examination that was used and validated by the Longitudinal Aging Study in India [8]. Other adaptations included printing out figures that needed to be copied on paper and having respondents copy the drawing below it, reducing challenges related to screen time-outs, poor vision due to screen light refraction, and other distractions [19]. The LOSHAK team worked closely with investigators from the Kenya Life Panel Study (KLPS) [20, 21] to adapt, translate, and pilot test instruments [8] to preserve consistency and comparability across studies. Gross et al. (2025) outline test adaptations implemented in KLPS and subsequently in LOSHAK, and Rianga et al. (2025) provide a comprehensive review of LOSHAK participant and researcher experiences with the assessments [19, 21]. 

Cognitive test items were conceptually organized into four domains: orientation, memory, executive function, and language (see Additional File 2, Supplementary Table S1). The orientation domain consisted of 10 questions related to time (month, year, day of week, day of month, and season) and place (country, city, name of nearby school, floor, and address). The memory domain included well-established tests, including the East Boston Memory Test (Brave Man) immediate recall [22], 10-word immediate and delayed recall [23, 24], and 3-word immediate and delayed recall [25, 26]. Executive functioning was assessed using backward day naming [26], serial 7 s [25], mental calculation for making change [25], and three-step instruction [26]. The language domain was comprised of animal naming, object identification (watch and pencil), and phrase repetition *(“Tupe tupate tumpatie Taak/Give us*,* we get it*,* so we give it to Taak”)* in Swahili. The tests were mapped a priori to various cognitive domains (Additional File 2, Supplementary Table S1) based on recognized theories, and existing factor analyses confirm the fit of these tests within each cognitive domain [27, 28]. 

Four psychosocial domains—depressive symptoms (Centre for Epidemiological Studies Depression Scale (CES-D)) [29], UCLA loneliness (3-item scale) [30], subjective well-being (Control, Autonomy, Self-Realization and Pleasure (CASP-19)) [31], and life satisfaction (Satisfaction with Life Scale) [32, 33] were measured using validated measures that have been used in other HRS family studies (Additional File 2, Supplementary Table S2).

### Analysis

We used means with standard deviations and counts with proportions to characterize distributions of the cognitive and psychosocial items for each cognitive and psychosocial variable. As an indication of the feasibility of administering the questions and whether participants were able to understand the questions, we evaluated missing data for each item. To describe internal consistency reliability for each cognitive and psychosocial domain, we calculated McDonald’s omega (ω) [34]. For each domain, confirmatory factor analysis models consistent with graded-response or continuous-response item response theory models were estimated, from which factor loadings and thresholds/intercepts were extracted. We hypothesized salient latent factors for memory, language, executive functioning, visuospatial functioning, and orientation based on neuropsychological theories [28]. Factor loadings describe the correlation of each item-level measure with other items, and item thresholds (for categorical items) or intercepts (for continuous items) provide information about the relative locations of items along the underlying latent cognitive or psychosocial trait [35]. To assess the marginal reliability of each cognitive and psychosocial confirmatory factor analysis model, we calculated the standard error of measurement [36]. Jones and colleagues (2024) provide a detailed overview of the confirmatory factor analysis models [37]. 

Model fit was assessed using standard global fit indices following prior research [7, 26, 38], including the Root Mean Square Error of Approximation (RMSEA), Comparative Fit Index (CFI), and Standardized Root Mean Residual (SRMR). Model fit was considered perfect if CFI = 1 and RMSEA = 0 and SRMR = 0, good if CFI ≥ 0.95 and RMSEA ≤ 0·05 and SRMR ≤ 0·05, adequate if CFI ≥ 0·90 and RMSEA ≤ 0·08 and SRMR ≤ 0·08, and poor if either CFI < 0·9 or RMSEA > 0·08 or SRMR > 0·08 [7]. To improve fit of the domain models, when necessary, we added theory-based residual correlations between items to accommodate empirical correlations among items greater than the model-estimated correlation explained by the factor [28]. Naming of common objects by sight (watch and pencil) within the language domain was allowed to be correlated with each other. Similarly, correlations were permitted among subjective well-being items related to control (Items 1 and 2), social connectedness (Items 12, 13, and 14), and decision-making freedom (Items 5 and 7) (see Additional File 2, Supplementary Table S2). Finally, the relatively small sample size of this pilot study precluded testing for systematic measurement differences by demographic characteristics (e.g., place of residence, education). Descriptive statistics were calculated using Stata statistical software, version 17 [39]. Factor analyses were conducted using Mplus version 8.7 [40].

## Results

Table 1 describes select demographic and socioeconomic characteristics of the LOSHAK pilot sample. Out of 203 respondents, the majority of the sample was female (58%). Most respondents were currently married (67%), and the average household included 6.4 members. 50% of the sample lived in rural areas, and the other half in peri-urban regions. Only 46% of the respondents were literate. In terms of schooling, 9% of the respondents had attended secondary school or above, while 41% attended primary school, and 40% had never attended school. Approximately one-third of the sample was still employed. Most households did not have access to electricity (55%), and on average, the number of rooms in the household was 2.7. Three-fourths of the households also owned livestock.

**Table 1:** Descriptive statistics of the LOSHAK, *N* = 203

	Mean (SD)/*N* (Percent)
Age	63.8 (11.5)
Male	85 (41.9%)
Rural residence	103 (50.7%)
Marital status	
Married	136 (67.0%)
Widowed	35 (17.2%)
Divorced/Single	11 (5.4%)
Missing marital status	21 (10.3%)
Household size	6.4 (3.2)
Literacy	
Literate	93 (45.8%)
Illiterate	108 (53.2%)
Missing information	2 (1%)
Schooling	
Did not attend any school	81 (39.9%)
Primary schooling	83 (40.9%)
Secondary schooling or above	18 (8.9%)
Missing schooling information	21 (10.3%)
Currently employed	75 (36.9%)
Average number of household rooms	2.7 (2.1)
Has electricity in the household	91 (44.8%)
Owns livestock	155 (76.4%)
Currently participates in Social Welfare Program	35 (17.2%)

Note: Social welfare programs refer to pension programs, cash transfers, relief food support, or the COVID-19 Economic Relief Plan

Across all cognitive tests, *N* = 39 participants (19%) were missing at least one cognitive test item. For most individual questions in the cognitive test battery, missing observations were present among < 10% of the sample, except for Serial 7 s, which had 30 missing observations (14.8%). Among the psychosocial variables, only the CES-D scale questions had missing observations, but missingness was under 7% for each item.

Table 2 provides McDonald’s omega values that describe the internal consistency reliability of each scale. Higher values indicate better reliability. McDonald’s omega for each cognitive domain was above 0.75, and for each psychosocial domain, it was above 0.83. These results suggest acceptable internal consistency for all domains for purposes of population-based research [41]. 

**Table 2:** Model fit statistics for cognitive and psychosocial domains, results from LOSHAK, *N* = 203

Domain	Number of items in the domain	% Total variance explained by first component from PCA	McDonald’s Omega	RMSEA	CFI	SRMR
Cognitive						
Orientation	10	35.50%	0.95	0.05	0.97	0.11
Memory	5	48.30%	0.79	0.07	0.99	0.03
Executive functioning	4	41.90%	0.75	0.00	1.00	0.03
Language/fluency	4	51.10%	0.87	0.00	1.00	0.03
Psychosocial						
Depressive symptoms	10	32.90%	0.83	0.10	0.93	0.06
Loneliness	3	76.00%	0.93	0.00	1.00	0.00
Subjective well-being	19	33.20%	0.92	0.09	0.90	0.08
Life satisfaction	5	65.70%	0.87	0.20	0.97	0.03

Note: Summary statistics in this table describe the suitability of the chosen model for each domain. Percentages of total variance explained come from Principal Components Analysis (PCA). McDonald’s Omega is an index of internal consistency reliability calculated from confirmatory factor analysis. Fit statistics (RMSEA, CFI, SRMR) describe the degree to which the model reflects the empirical correlations among the variables

Note:* RMSEA* Root Mean Square Error of Approximation, *CFI* Comparative Fit Index, *SRMR* Standardized Root Mean Residual

### Results from confirmatory factor analyses for cognitive domains

A single component explained between 35% and 51% of the total variance of items in each cognitive domain (Table 2). The fit of the confirmatory factor analysis models of cognitive function varied by domain. For the language domain, we included an additional or residual correlation between the two object-naming items – watch and pencil. We judged fit to be poor for orientation based on the SRMR (RMSEA:0.054, CFI:0.973, SRMR:0.110), adequate for memory (RMSEA:0.073, CFI:0.986, SRMR:0.031), good for executive functioning (RMSEA:0, CFI:1, SRMR:0.31), and good for language/fluency (RMSEA:0.00, CFI:1.00, SRMR:0.028) (Table 2).

Table 3 shows standardized factor loadings for each item in the four cognitive domains. We considered a good pattern of standardized factor loadings from a given model to have a range between 0.3 and 0.9 [34]. For the orientation domain, factor loadings ranged from 0.64 to 0.90, suggesting relatively consistent intercorrelations among items. For the memory domain, factor loadings ranged from 0.59 to 0.78, while for the executive function domain, there was a wider range from 0.40 to 0.92. The lower values for some items were still within the range that suggested meaningful intercorrelations with other items. For the language domain, standardized factor loadings ranged from 0.56 to 0.82. To improve the fit of the model for language, we added residual covariances between the confrontational naming items (watch and pencil).

**Table 3:** Factor loadings of cognitive items from confirmatory factor analysis models: results from LOSHAK (*N* = 203)

Variable	Factor Loadings
Orientation	
Day of month	0.875
Month	0.891
Year	0.896
Day of the week	0.764
What county are we in	0.809
What city/village are we in	0.842
Season of year	0.691
Floor of building	0.74
Address (street name and/or building number)	0.638
Name of the nearest school	0.848
Memory	
CERAD immediate sum of 3 trials	0.778
Three-word delayed recall	0.605
CERAD word list delay	0.654
Brave Man Immediate (East Boston Memory Test)	0.589
Three-word immediate registration	0.666
Executive functioning	
Backward Day naming	0.916
Serial 7s	0.641
Making change	0.633
Three-step instruction	0.398
Language	
Animal fluency	0.703
Object naming (watch)	0.823
Object naming (pencil)	0.815
Repetition of a phrase	0.560

Supplementary Figure S1 (Additional File 2) graphically illustrates item thresholds for each cognitive domain. These maps are based on parameters from the confirmatory factor analyses and display the same loadings as seen in previous tables, but they also include item thresholds for each item within a domain. The item thresholds, reflecting relative locations along each latent trait, which are each scaled to an N(0,1) distribution, are between − 2 and 0.5 SD along the latent factor for the orientation domain, indicating that some orientation items tend to be answered correctly by most of the sample, as expected based on prior studies [7]. The thresholds were between − 2.5 and 2.5 for memory, −1.5 and 1.5 SD for executive functioning, and between − 2.5 and 2 SD for the language domain.

**Fig. 1:**
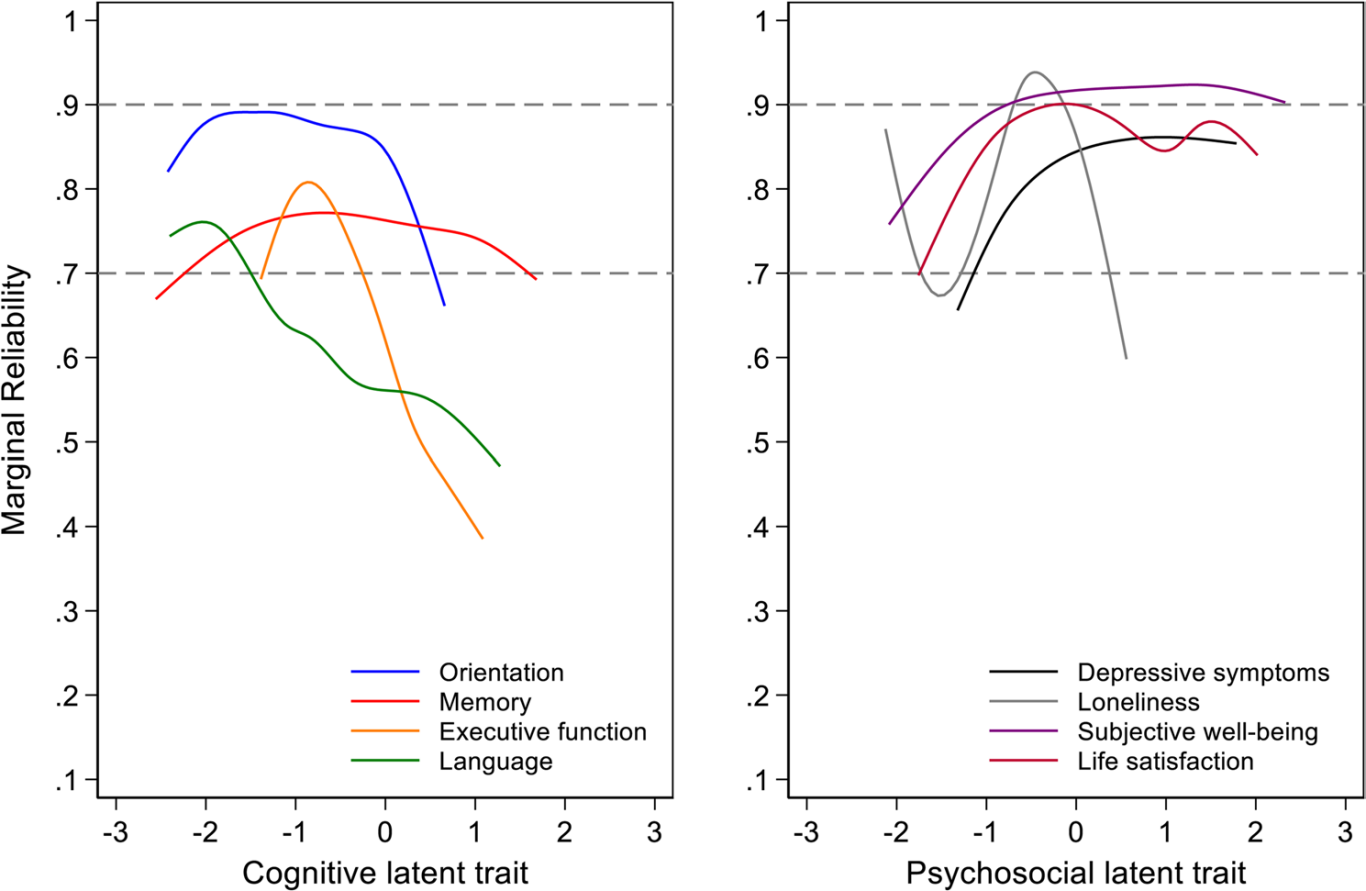
Model-estimated marginal reliability for cognitive and psychosocial domains: Results from LOSHAK (*N* = 203) Note: These plots show the marginal reliability of measurement models for each domain. This figure illustrates differences in the reliability of estimated factor scores over levels of the latent trait

Figure 1 (left panel) shows marginal reliabilities based on the confirmatory measurement model across each cognitive domain and over the range of each latent trait. The reliability of the measurement model is above *r*= 0.7, an accepted cutoff for population-based research [41], for 59%, 100%, 39%, and 4% of participants for orientation, memory, executive functioning, and language, respectively. These differences in marginal reliability reflect the number and level of difficulty of items in each domain.

### Results from confirmatory factor analyses for psychosocial domains

For the psychosocial measures, based on principal component analyses, a single component explained between 33% and 76% of the total variance of items in each psychosocial domain (Table 2). Fit of confirmatory factor analysis models was adequate for depressive symptoms (RMSEA:0.095, CFI:0.934, SRMR:0.064), poor for subjective well-being (RMSEA:0.093, CFI:0.904, SRMR:0.081), perfect for loneliness because the model was fully saturated (RMSEA:0, CFI:1, SRMR:0), and poor for life satisfaction (RMSEA:0.20, CFI:0.97, SRMR:0.03) (Table 3). To improve the fit of the model for subjective well-being, we added residual covariances between items 1 and 2; items 5 and 7; and items 12, 13, and 14.

The factor loadings generally suggested adequate intercorrelations (Table 4). In the depressive symptoms domain, one item had a negative factor loading, indicating a translation problem and standardized factor loadings for other items ranged from 0.33 to 0.79. Factor loadings for the loneliness domain were high and ranged from 0.82 to 0.91; for subjective well-being, they ranged from 0.31 to 0.82; and for life satisfaction, the range was from 0.63 to 0.91. Figure S2 (Additional File 2) graphically illustrates item thresholds for each psychosocial domain. Thresholds for psychosocial domains ranged between − 1.5 and 2.5 SD for depressive symptoms, −3 and 0 SD for loneliness, −2 and 2.5 SD for subjective well-being, and − 1.5 and 0.5 SD for life satisfaction.

**Table 4:** Factor loadings of psychosocial items from confirmatory factor analysis models: results from LOSHAK (*N* = 203)

Variable	Factor loadings
Depressive symptoms	
Item 1: Bothered by things	0.655
Item 2: Had a problem concentrating	0.684
Item 3: Felt depressed and troubled	0.788
Item 4: Everything took energy	0.736
Item 5: Felt hopeful about the future *	−0.057
Item 6: Felt afraid	0.737
Item 7: Difficulty sleeping peacefully	0.615
Item 8: Were happy *	0.328
Item 9: Felt lonely	0.602
Item 10: Lacked the motivation to do anything	0.450
Loneliness	
Item 1: Feel lack of companionship	0.821
Item 2: Feel left out	0.973
Item 3: Feel isolated	0.914
Subjective well-being	
Item 1: My age prevents me from doing the things*	0.672
Item 2: I feel that what happens to me is out of my control	0.582
Item 3: I feel free to plan for the future	0.648
Item 4: I feel left out of current activities/happenings*	0.356
Item 5: I can do activities that I want to do	0.576
Item 6: Family responsibilities prevent me from doing what I want to do*	0.41
Item 7: I feel that I can do activities as I please	0.741
Item 8: My health stops me from doing things I want to do*	0.569
Item 9: Shortage of money stops me from doing things I want to do*	0.216
Item 10: I look forward to each day	0.738
Item 11: I feel that my life has value/purpose	0.822
Item 12: I enjoy the activities that I do	0.685
Item 13: I enjoy being in the company of others	0.505
Item 14: On balance, I look back on my life with a sense of happiness	0.305
Item 15: I feel quite vibrant these days	0.726
Item 16: I choose to do activities that I have never done before	0.643
Item 17: I feel satisfied with the way my life has turned out	0.490
Item 18: I feel that life is full of opportunities	0.813
Item 19: I feel that the future looks good for me	0.781
Life satisfaction	
Item 1: In most ways my life is close to ideal	0.719
Item 2: The conditions of my life are excellent	0.740
Item 3: I am satisfied with my life	0.910
Item 4: So far, I have got the important things I want in life	0.770
Item 5: If I could live my life again, I would change almost nothing	0.631

*Reverse-coded

Figure 1 (right panel) shows marginal reliabilities for confirmatory measurement models of each psychosocial domain. Reliability of the measurement model is above *r* = 0.7 for 100%, 52%, 99%%, and 100% of participants for depressive symptoms, loneliness, subjective well-being, and life satisfaction, respectively.

## Discussion

The overarching goal of LOSHAK is to build the infrastructure necessary to collect robust data on key domains related to aging, promoting the health and economic well-being of a nationally representative sample of older adults in Kenya. Although the goal of the pilot phase of LOSHAK was to gauge acceptability, results from this study’s psychometric analyses additionally suggest the cognitive and psychosocial surveys were translated, administered, and collected well. This contributes to the growing body of literature demonstrating the feasibility of collecting high-quality data in LMICs, provided rigorous interviewer training and quality checks [21, 26, 42, 43]. The pilot/feasibility phase of LOSHAK provides an example of how to adapt tools and make them culturally and linguistically appropriate. Our study’s findings provide a roadmap for future studies to consider when adapting instruments from the HRS and HCAP initiatives.

We examined the factor structure of cognitive and psychosocial tests administered in the LOSHAK pilot sample. This is an important exercise because most existing cohorts with such available data have been conducted in high-income Western populations, with tests predominantly administered in English. Summarizing performance across multiple cognitive tests that reflect common underlying domains provides a structured representation of cognitive and psychosocial functioning. Results from the present pilot study demonstrate that collecting cognitive and psychosocial measures in Kenya, with appropriate contextual adaptation, is both feasible and informative. These findings are consistent with previous work in other countries [7, 21], and suggest cognitive and psychosocial test items cluster into a priori domains as expected [26, 37, 44]. This finding supports the construct validity of these instruments for measuring their target constructs in a Kenyan population, as well as cross-context applicability of a shared cognitive measurement framework across international aging studies [7]. The high values of McDonald’s Omega in this study speak to the internal consistency of cognitive and psychosocial measures adapted for the Kenyan context. Further, a single factor in CFAs accounted for 35–51% of the variance in cognitive domains and 33–76% in psychosocial domains, with good fit observed for memory and executive functioning. Distributions of item thresholds indicate that some domains, namely orientation and loneliness, included relatively easy items, while others measure a broader range along the latent trait. Marginal reliability was above 0.7 for the majority of participants in memory, depressive symptoms, subjective well-being, and loneliness domains, but was lower for orientation, executive functioning, and language.

Low levels of item missingness suggest participants were engaged throughout the interview, and moreover, that the goal of population representativeness in a larger, national project can be met. Although the proportion of participants with missing values across any cognitive test was 19%, missingness on individual tests was < 10%. This level of missingness is feasible because non-missing data for some items can be used to inform missingness in other items. A generally accepted rule of thumb is that when the missingness is < 5% on a given variable, the risk of bias is ignorable [45], whereas levels of missingness above 10% might introduce more substantial risk [46]. An important future next step is to evaluate the diagnostic accuracy of this robust cognitive test battery, adapted for the Kenyan context, in distinguishing dementia, mild cognitive impairment, and cognitively normal individuals, using a larger planned sample than the current pilot.

Strengths of this study include careful translation to Swahili (the local language in Kilifi, Kenya) and contextual adaptation of instruments. This was undertaken in collaboration with the KLPS study, so that both Kenyan studies were able to collect harmonizable data to characterize late-life health and economic well-being across LOSHAK’s future population-based sample and KLPS’ causal framework [20], and interviewers underwent extensive training and certification [19]. We also note several limitations. The pilot study is not nationally representative, however future waves will include a larger, geographically diverse sample that will be nationally representative. The study results also highlight areas for improvement that need to be addressed as the study scales. The higher share of missingness for the Serial 7 s question may be a reflection of the poor levels of numeracy or issues with question format and position. The poor fit of the psychosocial domains, especially for depressive symptoms and life satisfaction, also warrants further investigation since issues due to translations, interpretation of existing questions, and cultural/societal relevance are likely when an instrument is administered in a distinct context.

## Conclusion

Identification of factors associated with ADRD prevalence and cognitive change among older adults is increasingly important in Kenya as the older population grows [11, 12]. Population-based panel studies, like the HRS in the US and LOSHAK in Kenya, have great potential to improve our understanding of social, economic, and health factors that impact the health and financial well-being of people and society. This LOSHAK pilot study provides a foundation for collecting nationally representative data in Kenya, as has been done in numerous other countries, where harmonized HRS and HCAP studies [26] have been implemented.

## Supplementary Information

Additional File 1. The LOSHAK pilot questionnaire.

Additional File 2. Provides summary statistics (mean, standard deviation, minimum and maximum values) for each item in the cognitive and psychosocial domains. It also includes the item threshold plots for each domain. The table of contents is listed below: Supplementary Table S1: Statistics for cognitive domains: Results from LOSHAK (*N*=203). Supplementary Table S2: Statistics for psychosocial domains: Results from LOSHAK (*N*=203). Supplementary Figure S1: Person-plot of item thresholds linked to the latent Cognition, Cognitive domains: Results from LOSHAK (*N*=203). Supplementary Figure S2: Person-plot of item thresholds, Psychosocial domains: Results from LOSHAK (*N*=203).

## Data Availability

The data will be available on the Center for Global Health Equity (University of Michigan) website shortly.
