# Supplementary material for: Validation of cognitive and psychosocial tools in Kenya: findings from the LOSHAK feasibility pilot
Source: BMC Public Health. 2025 Nov 17;25:4008. doi: 10.1186/s12889-025-24918-z (PMC12625501; doi:10.1186/s12889-025-24918-z)
Supplement: Supplementary file 1 — Additional File 1. The LOSHAK pilot questionnaire. [file 12889_2025_24918_MOESM1_ESM.pdf]

Longitudinal Study of Health and Ageing in Kenya (LOSHAK)

Version: December 16, 2022 — ENGLISH

SECTION 1: PRE-INTERVIEW INFORMATION

Fill in this information before the interview begins:

PI_0001. Identification Number of Focus Respondent (Sample Person's (SP's)) KRHDSS permanent ID): |___|___|___|___|___|___|___|

PI_0002. Sample Person's(SP) Family Name:
____

PI_0003. SP's (a) Name 1 / (b) Name 2: (a)______________________ / (b)
____

PI_0004. SP's Gender: |____| (1=Male, 2=Female)

PI_0005. Date of interview: (DD/MM/YYYY)
|___|___|/|___|___|/|___|___|___|___|

PI_0006. Time start interview: (24 hr clock)

|___|___| : |___|___|

PI_0007. Enumerator ID: |___|___|___|___|

PI_0008. Enumerator's name: (first) _____________________ / (surname)
____

PI_0009. Household information (HH):

a. Size of HH –

b. Number of children/adults –

c. Head of household's name –

d. Head of household's age -

SECTION 2: READ INFORMED CONSENT (IC)

IC_0001. ***Write <u>your</u> (enumerator's) name to indicate SP's acceptance:***

____

IC_0002. *[**ENUMERATOR NOTE**: DO NOT READ. SIMPLY RECORD YOUR IMPRESSIONS]*
Is the respondent able to proceed with the survey?

1 = Yes-SP agrees to participate

2 = No-SP refuses to participate

3 = No-SP does not refuse but is unable to participate)

If YES, skip to Section 3. If NO, continue.

IC_0002a. *[**ENUMERATOR NOTE**: DO NOT READ. SIMPLY RECORD YOUR IMPRESSIONS]*

1 = Wants to reschedule ***(skip to "Rescheduling instructions" below)***

2 = Refusal for this round only ***(skip to question IC_0002b)***

3 = Refusal for this round and any future rounds ***(skip to question IC_0002c)***

4 = Unable to survey – spouse refusal ***(skip to "Closing Interview Statement")***

5 = Unable to survey – contact or other relative refusal ***(skip to "Closing Interview Statement")***

6 = Unable to survey – in prison ***(end interview)***

7 = Unable to survey – mental illness / disability ***(skip to question IC_0003)***

10 = Unable to survey – other ***(skip to question IC_0002d)***

Rescheduling instructions: Please ask the SP when they are next available, and then call your team lead (or other senior team member) to confirm this day and time. If you are unable to confirm this day and time, make a tentative appointment with the SP. Then, let the SP know that you will contact them to confirm when you will return. Record this information and the current time on the tracking sheet now. End the interview.

IC_0002b. ***Record your impressions of why the SP refuses to participate during this survey round. If you feel comfortable doing so, you may ask theSP why:*** Why don't you want to participate? ***Choose up to 3 reasons*** (Instructions for CAPI: Multiple answers allowed)

1 = Survey is too long

2 = SP has caregiving duties

3 = SP has to work

4 = SP does not want to disclose personal information

5 = SP is suspicious of enumerator

6 = SP hasn't received (further) assistance from Aga Khan University and doesn't want to participate because of that

7 = SP just doesn't want to / no reason given

10 = Other (specify)

Skip to "Closing Interview Statement".

IC_0002c. ***Record your impressions of why the SP refuses to participate during this round and any future rounds. If you feel comfortable doing so, you may ask the SP why:*** Why don't you want to participate?

____

Skip to "Closing Interview Statement".

IC_0002d. ***Record your impressions of why we are unable to survey the respondent during this round.***

____

Read: Thank you very much for your time. ***End interview here.***

IC_0003. ***Record your impressions of the SP's disability. If you feel comfortable doing so, ask the SP or the SP's caretaker:*** What is his/her disability?

____

The following questions collect some basic information for a SP who is mentally ill or disabled. Ask these questions of the SP or the SP's caretaker.

IC_0003a. What is the highest level of education he/she attended? ________

IC_0003b. What is his/her occupation? ________

IC_0003c. Is he/she married?

1 = Yes

2 = No

Read: Thank you very much for your time. ***End interview here.***

Closing Interview Statement. Read: Thank you very much for your time. If you change your mind and would like to participate in the interview, please contact us at the Aga Khan University office. ***End interview here.***

SECTION 3: SELF-REPORTED COGNITIVE ABILITIES

SRC_0001. Part of this study is concerned with people's memory and ability to think about things. First, how would you rate your memory at the present time? Would you say it is:

1 = Very good

2 = Good

3 = Average

4 = Poor

5 = Very poor

SRC_0002. Compared to two years ago, would you say your/SP's memory is:

1 = Better now

2 = About the same

3 = Worse now than it was then

SRC_0003. How would you rate your/SP's other mental abilities such as thinking clearly and solving problems at the present time? Would you say they are:

1 = Very good

2 = Good

3 = Average

4 = Poor

5 = Very poor

SRC_0004. Compared to two years ago, would you say your/SP's other mental abilities are:

1 = Better now

2 = About the same

3 = Worse now than it was then

SECTION 4: COGNITION

Section 4.1 - Swahili Mental State Exam (SMSE)

[READ] *In the next section, I will ask questions and have you do simple things to check how your brain is functioning. Sometimes it is hard, don't worry. Answer the questions the best you can. You have 10 seconds for most questions.*

*[**ENUMERATOR NOTE:** IF THE RESPONDENT DOESN'T UNDERSTAND THE QUESTION, TRY TO CLARIFY/REPHRASE (UNLESS OTHERWISE NOTED IN THE INSTRUCTIONS). ALSO, ONLY SELECT "DON'T KNOW" WHEN THE RESPONDENT REALLY DOESN'T KNOW, AND NOT BECAUSE THEY DON'T UNDERSTAND THE WORDING. IF THEY DON'T UNDERSTAND THE WORDING, THEN TRY TO REPHRASE/CLARIFY (UNLESS OTHERWISE NOTED).]*

*[**ENUMERATOR NOTE:** BEFORE BEGINNING THE SURVEY, MAKE SURE YOU KNOW THE NAME OF THE VILLAGE YOU ARE IN AND THE LOCATION OF LOCAL MARKETS SO THAT YOU CAN VERIFY THE SP'S RESPONSES.]*

SMSE_1. What year is it? *[**ENUMERATOR NOTE:** PROBE IF THE RESPONDENT DID NOT INTERPRET THE QUESTION CORRECTLY].*
0=Incorrect response | 1=Correct response | -77= Don't know | -88=Refused

SMSE_2. Which season of the year is this? *[**ENUMERATOR NOTE:** ACCEPTABLE ANSWERS INCLUDE LONG RAIN OR SHORT RAIN SEASON, HOT OR COLD SEASON. PROBE IF THE RESPONDENT DID NOT INTERPRET THE QUESTION CORRECTLY (E.G., WEATHER SEASON)]*
0=Incorrect response | 1=Correct response | -77= Don't know | -88=Refused

SMSE_3. What month is it?
0=Incorrect response | 1= Correct response | -77= Don't know | -88=Refused

SMSE_4. What is today's date?
0= Incorrect response | 1=Correct response | -77= Don't know | -88=Refused

SMSE_5. What day of the week is it?
0=Incorrect response | 1=Correct response | -77= Don't know | -88=Refused

SMSE_6. What county are we in right now?
0=Incorrect response | 1=Correct response | -77= Don't know | -88=Refused

SMSE_7. What is the name of this city/town/village?
0=Incorrect response | 1=Correct response | -77= Don't know | -88=Refused

SMSE_8. What is the name of this city/town/village?

0=Incorrect response | 1=Correct response | -77= Don't know | -88=Refused

SMSE_9. What is your physical address?

0=Incorrect response | 1=Correct response | *-77= Don't know* | *-88=Refused*

SMSE_10. What room are we in?

0=Incorrect response | 1=Correct response | *-77= Don't know* | *-88=Refused*

SMSE_11. Now I am going to tell you the names of three objects and I would like you to repeat them back to me. Are you ready? Let's begin.

a.	b.	c.
*[**Enumerator Note:** SAY THE FOLLOWING WORDS SLOWLY AT 1-SECOND INTERVALS]*	I will repeat the names of the three objects again. Once I am done, please repeat them back to me.<br><br>*[**Enumerator Note:** SAY THE FOLLOWING WORDS SLOWLY AT 1-SECOND INTERVALS]*	I will now repeat the names of the three objects one last time. Once I am done, please repeat them back to me.<br><br>*[**Enumerator Note:** SAY THE FOLLOWING WORDS SLOWLY AT 1-SECOND INTERVALS]*
Car	Car	Car
House	House	House
Fish	Fish	Fish
SMSE_11a Now please repeat them back to me.<br><br>***[DO NOT READ]*** *HOW MANY WORDS DID THE RESPONDENT REPEAT CORRECTLY?*	SMSE_11b Now please repeat them back to me.<br><br>***[DO NOT READ]*** *HOW MANY WORDS DID THE RESPONDENT REPEAT CORRECTLY?*	SMSE_11c Now please repeat them back to me.<br><br>***[DO NOT READ]*** *HOW MANY WORDS DID THE RESPONDENT REPEAT CORRECTLY?*

0=None| 1=1 Correct | 2= 2 Correct | 3= 3 Correct<br><br>*[Enumerator note: IF 11a=3 -> Go to SMSE_12 If 11a=0 OR 1 OR 2 -> Got to 11b]*	*0=None | 1=1 Correct | 2= 2 Correct | 3= 3 Correct*<br><br>*[Enumerator note: IF 11b=3 -> Go to SMSE_12 If 11b= 0 OR 1 OR 2 -> Go to Q11c]*	*0=None | 1=1 Correct | 2= 2 Correct | 3= 3 Correct*

SMSE_11d Calculate the number of trials until all three words are recalled successfully _______

READ: Very good, now try to remember these words because I will ask you about them later.

SMSE_12. **READ:** Now I'd like you to subtract 7 from 100. Then keep subtracting 7 from each answer until I tell you to stop. Let's start with the first subtraction.

*[**ENUMERATOR NOTE:** RECORD THE PARTICIPANT'S RESPONSES. SCORE THE TOTAL NUMBER OF CORRECT ANSWERS. CORRECT SUBTRACTIONS ARE 7 LESS THAN THE PREVIOUS NUMBER GIVEN.]*

a. Number 1	b. Number 2	c. Number 3	d. Number 4	e. Number 5
What is 100 take away 7?<br><br>Ans__	Now what is {Number 1} take away 7?<br><br>Ans___	Now what is {Number 2} take away 7?<br><br>Ans___	Now what is {Number 3} take away 7?<br><br>Ans___	Now what is {Number 4} take away 7?<br><br>Ans___

You can stop now.

SMSE_13. Please name the days of the week in backwards order starting from Sunday. For example, before Sunday comes Saturday. What comes before that?

a . Day 1	***b. Day 2***	***c. Day 3***	***d. Day 4***	***e. Day 5***
1= Correct- Friday/ 0= Incorrect	*1= Correct - Thursday/ 0= Incorrect*	*1= Correct - Wednesday/ 0=Incorrect*	*1 = Correct - Tuesday 0=Incorrect*	*1 = Correct - Monday/ 0=Incorrect*

SMSE_14 A few minutes ago, I asked you to remember the names of three objects. Now what were the three objects that I asked you to remember?
0= None correct | 1= 1 Correct | 2= 2 Correct | 3= 3 Correct | -88=Refused

I will now point to different objects and I would like you to tell me the name of the object.

SMSE_15 **[ENUMERATOR NOTE:** POINT TO WATCH]
What is this called?
[**ENUMERATOR NOTE:** CORRECT ANSWER IS WATCH]
0= Incorrect response | 1= Correct response | -77= Don't know | -88=Refused

SMSE_16 **[ENUMERATOR NOTE:** SHOW PENCIL]
What is this called?
[**ENUMERATOR NOTE:** CORRECT ANSWER IS PENCIL]
0= Incorrect response | 1= Correct response | -77= Don't know | -88=Refused

SMSE_17 Now I would like you to repeat what I say: 'Tupe tupate tumpatie Taaka.'
***[ENUMERATOR NOTE:** THE RESPONDENT IS ALLOWED ONLY ONE ATTEMPT TO REPEAT THE PHRASE. THE INTERVIEWER CANNOT REPEAT THE PHRASE IF THE RESPONDENT HAS ALREADY ATTEMPTED THE PHRASE. IF THE RESPONDENT STRUGGLES TO HEAR THE PHRASE, THE INTERVIEWER CAN REPEAT THE PHRASE UP TO FIVE TIMES.]*
[**DO NOT READ:** Did the respondent repeat the phrase correctly?]
0= Incorrect response | 1= Correct response | -77= Don't know | -88=Refused

SMSE_17a. Can you read?
0 = No | 1 = Yes | -77= Don't know | -88=Refused

SMSE_17b. Can you write?
0 = No | 1 = Yes | -77= Don't know | -88=Refused

[Instructions for CAPI: If SMSE_17a=1, the next question to pop-up must be SMSE_18a. If SMSE_17a=0, skip to SMSE_18b]

SMSE_18a. **[If SMSE_17a = 1]** This next part involves a task where I ask you to read the bolded words that are on the screen and follow the instructions. Are you ready? Let's begin.

*[**ENUMERATOR NOTE**: HAND THE PERSON THE TABLET WITH '**CLOSE YOUR EYES**' ('**FUNGA MACHO**') ON IT. IF THE SUBJECT READS AND DOES NOT CLOSE THEIR EYES, RE-READ THE INSTRUCTIONS. THIS CAN BE REPEATED UP TO THREE TIMES. SCORE ONLY IF THE SUBJECT CLOSES THEIR EYES.]*

[Instructions for CAPI: print 'FUNGA MACHO' large so it fills entire tablet screen]

[**DO NOT READ:** Did the respondent follow the instructions?]
0 = Doesn't close their eyes | 1 = Closes their eyes | -88= Refused
You can open your eyes now.

SMSE_18b. **[If SMSE_17a = 0]** This next part involves a task where I will do something and ask you to copy what I do. Please observe and copy what I do. *[**ENUMERATOR NOTE**: CLOSE YOUR EYES, THEN OPEN THEM. PLEASE KEEP YOUR EYES CLOSED FOR 1-2 SECONDS. IF THE RESPONDENT DOES NOT CLOSE THEIR EYES, REPEAT THE INSTRUCTIONS AND CLOSE AND OPEN YOUR EYES AGAIN. THIS CAN BE REPEATED UP TO THREE TIMES. SCORE ONLY IF THE SUBJECT CLOSES THEIR EYES.]*
[**DO NOT READ:** Did the respondent follow the instructions?]
0= Doesn't close their eyes | 1= Closes their eyes | -88= Refused
You can open your eyes now.

SMSE_19a. Are you right- or left-handed?
1= Right-handed | 2 = Left-handed

SMSE_19b*. [**ENUMERATOR NOTE**: TAKE A PIECE OF PAPER AND HOLD IT UP IN FRONT OF THE PERSON. PLEASE READ THE INSTRUCTIONS COMPLETELY AND HAND THEM THE PIECE OF PAPER AFTERWARD. THE INTERVIEWER CAN READ THE INSTRUCTIONS ONLY ONCE. THE INTERVIEWER CAN REPEAT THE INSTRUCTIONS ONLY IF THE RESPONDENT DID NOT HEAR THE INSTRUCTIONS.]*
Next I'm going to give you a piece of paper. When I do, I would like you to do the following: take the paper in your right/left hand, fold the paper in half with both hands, and put the paper down on your lap.
[**DO NOT READ:** Did the respondent follow the instructions (mark all that apply)?]
0= None | 1= Takes paper correctly in hand | 2= Folds it in half | 3= Puts it on his/her lap | -88=Refused

SMSE_20a*.* **[If SMSE_17b = 1]** Now, I am going to give you a pencil and a piece of paper. Please write any complete sentence on that piece of paper.
*[**Enumerator Note:** HERE THE RESPONDENT CAN WRITE ANYTHING AS LONG AS YOU CAN UNDERSTAND THE MEANING, AND IN ANY LANGUAGE HE OR SHE IS COMFORTABLE WITH. THE RESPONDENTS MAY HESITATE AND WOULD REFUSE TO DO IT. BUT YOU NEED TO ENCOURAGE THEM TO WRITE AT LEAST ONE SHORT SENTENCE. IF SENTENCE IS ILLEGIBLE, ASK "COULD YOU READ IT FOR ME?" SENTENCE SHOULD HAVE A SUBJECT AND A VERB, AND MAKE SENSE. SPELLING AND GRAMMATICAL ERRORS ARE ACCEPTABLE]*

[DO NOT READ]: Did the respondent write a complete sentence?
0= Incorrect response | 1= Correct response | -88=Refused

SMSE_20b. **[If SMSE_17b = 0]** Please tell us something about your house.
[DO NOT READ]: Did the respondent say a full sentence?

*[**ENUMERATOR NOTE**: THE SENTENCE MUST BE A FULL SENTENCE ABOUT HIS/HER HOUSE AND MUST MAKE SENSE.]*
0= Incorrect response | 1= Correct response | -88=Refused

*SMSE_21. [**ENUMERATOR NOTE:** PLACE DESIGN, ERASER AND PENCIL IN FRONT OF THE PERSON].*
Now I will show you a picture of shapes and ask you to copy the picture on the piece of paper in front of you.

[Instructions for CAPI: print interlocking pentagons large so that they fill entire tablet screen.]

*[**ENUMERATOR NOTE:** SHOW THE RESPONDENT THE PICTURE THE WHOLE TIME. THE RESPONDENT IS ALLOWED TO ERASE AND START OVER. WAIT UNTIL THE PERSON IS FINISHED AND HANDS IT BACK. SCORE ONLY FOR A CORRECTLY COPIED DIAGRAM WITH A 4-SIDED FIGURE BETWEEN TWO 5-SIDED FIGURES. GIVE ONE MINUTE. IF SP IS STILL WORKING AFTER 1 MINUTE, PLEASE NOTE THE FIGURE THAT WAS DRAWN AT ONE MINUTE]*

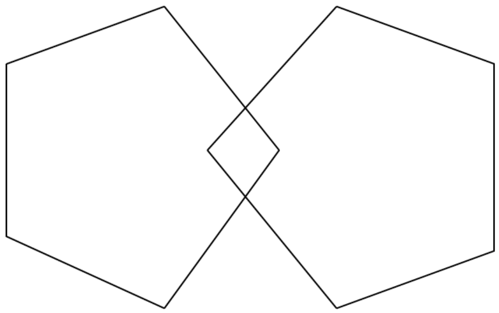

Copy here

*SMSE_21a. **[DO NOT READ]** DOES THE DRAWING HAVE TWO FIVE-SIDED FIGURES THAT INTERSECT TO FORM A FOUR-SIDED FIGURE?*
0= No | 1= Yes

*SMSE_21b. **[DO NOT READ]** DOES THE DRAWING HAVE ALL ANGLES IN THE FIVE-SIDED FIGURES PRESERVED?*
0=No | 1= Yes

Section 4.2 - 10-Word Recall

READ: *I am going to read out a list of words and ask you to repeat them back to me when I have finished. The list of words is purposely made long so that it will be difficult for anyone to recall all the words. You can repeat the words back in any order. Please listen carefully as I cannot repeat the words after I have read them to you.*

*CE_0001 [**ENUMERATOR NOTE:** READ OUT THE TEN WORDS, PAUSING FOR ONE SECOND BETWEEN EACH. TICK THE ORDER IN WHICH THE RESPONDENT SAYS WORDS AND RECORD ANY WORDS THAT ARE NOT ON THE LIST. RECORD ON A PIECE OF PAPER AND FILL IN SURVEY AFTER.]*

**1st**	**Order**
**MILK-**	
**ARM-**	
**LETTER-**	
**QUEEN-**	
**TICKET-**	
**GRASS-**	
**CORNER-**	
**STONE-**	
**BOOK-**	
**STICK-**	
**Other Words:**	
**Total Score**	

READ: Now please tell me all the words you can remember in any order.

[ENUMERATOR NOTE: SCORE TOTAL NUMBER OF WORDS CORRECTLY RECALLED IN THE BOX ABOVE UNDER COLUMN 2]

CE_0002 **READ:** Thank you. Now I will read out the words to you one more time. Again, please listen carefully, as I will ask you to repeat the words when I have finished.

[***ENUMERATOR NOTE:*** *READ OUT THE TEN WORDS, PAUSING FOR ONE SECOND BETWEEN EACH.*]

**2nd**	**Order**
**GRASS-**	
**TICKET-**	
**STICK-**	
**BOOK-**	
**MILK-**	
**CORNER-**	
**ARM-**	
**STONE-**	
**QUEEN-**	
**LETTER-**	
**Other Words:**	
**Total Score**	

READ: Now please tell me all the words you can remember

*[**ENUMERATOR NOTE:** SCORE TOTAL NUMBER OF WORDS CORRECTLY RECALLED IN THE BOX ABOVE UNDER COLUMN 2]*

CE_0003 **READ:** Thank you. Now I will read out the words to you one last time. Again, please listen carefully, as I will ask you to repeat the words when I have finished.

[***ENUMERATOR NOTE:*** *READ OUT THE TEN WORDS, PAUSING FOR ONE SECOND BETWEEN EACH.*]

3rd	Order
LETTER**-**	
GRASS**-**	
ARM**-**	
STONE**-**	
CORNER**-**	
QUEEN**-**	
MILK**-**	
BOOK**-**	
STICK**-**	
TICKET**-**	
Other Words	
Total Score	

READ: Now please tell me all the words you can remember

*[**ENUMERATOR NOTE**: SCORE TOTAL NUMBER OF WORDS CORRECTLY RECALLED IN THE BOX ABOVE UNDER COLUMN 2]*

CE_0004 [Do not read out loud] INDICATE WHETHER ANY OF THE FOLLOWING APPLY TO THE ADMINISTRATION OF THE WORD LISTS

1 = AN INTERRUPTION OCCURRED DURING ADMINISTRATION OF LIST 1

2 = AN INTERRUPTION OCCURRED DURING ADMINISTRATION OF LIST 2

3 = AN INTERRUPTION OCCURRED DURING ADMINISTRATION OF LIST 3

4 = RESPONDENT HAD DIFFICULTY HEARING THE WORDS

0= NO ISSUES OCCURRED

Section 4.3 - Animal Naming

READ: Now we are going to do something a little different. I am going to give you a category, and I want you to name, as fast as you can, all of the things that belong in that category. For example, if I say 'articles of clothing' you could say shirt, tie or hat. Can you think of other articles of clothing?

*[**ENUMERATOR NOTE:** WAIT FOR THE SUBJECT TO GIVE TWO WORDS. IF THE SUBJECT SUCCEEDS, INDICATE THAT THE RESPONSES WERE CORRECT AND PROCEED TO THE TEST ITSELF. IF THE SUBJECT GIVES AN INAPPROPRIATE WORD OR REPLY, CORRECT THE RESPONSE AND REPEAT THE INSTRUCTIONS. IF IT BECOMES CLEAR THAT THE SUBJECT STILL DOES NOT UNDERSTAND THE INSTRUCTION, TERMINATE THIS TASK AND EXPLAIN WHY THIS IS SO. AFTER YOU ARE SATISFIED THAT THE SUBJECT UNDERSTANDS THE TASK, AND HAS GIVEN TWO WORDS NAMING ARTICLES OF CLOTHING, PROCEED]*

READ: That's fine. I want you to name things that belong to another category, 'animals'. I want you to think about all the many different kinds of animals you know. Think of any kind of animal in the air, on land, in the water, in the forest, all the different animals. Now I would like for you to tell the names for as many different animals as you can. You will have a minute to do this. Are you ready? Let's begin.

*[**ENUMERATOR NOTE:** BEGIN TIMER. ALLOW ONE MINUTE PRECISELY. IF THE SUBJECT STOPS BEFORE THE END OF THE TIME, ENCOURAGE THEM TO TRY TO FIND MORE WORDS. IF THEY ARE SILENT FOR 15 SECONDS REPEAT THE BASIC INSTRUCTION*

- *('I WANT YOU TO TELL ME ALL THE ANIMALS YOU CAN THINK OF').*
- *('NATAKA UNIAMBIE WANYAMA WOTE AMBAO UNAWEZA KUFIKIRIA').*

NO EXTENSION ON THE TIME LIMIT IS MADE IN THE EVENT THAT THE INSTRUCTION HAS TO BE REPEATED.]

AN_0001 **[DO NOT READ]:** DID ANY ISSUES OCCUR DURING THE ADMINISTRATION OF THIS TASK?

0= No issues

1= Interruption during the response period

2= Problem with timing

3= Respondent did not understand the task

4= Another issue occurred

-88= Refused

AN_0002 **[DO NOT READ]:** ENTER THE NUMBER OF TOTAL ANIMAL ANSWERS

[ENUMERATOR NOTE: ANY MEMBER OF THE ANIMAL KINGDOM, REAL OR MYTHICAL IS SCORED CORRECT, EXCEPT REPETITIONS AND PROPER NOUNS. SPECIFICALLY, EACH OF THE FOLLOWING GETS CREDIT: A SPECIES NAME AND ANY 9 ACCOMPANYING BREEDS WITHIN THE SPECIES; MALE, FEMALE AND INFANT NAMES WITHIN THE SPECIES.]

AN_0003 [DO NOT READ]: ENTER THE NUMBER OF INCORRECT ANIMALS NAMED

AN_0004 [DO NOT READ]: ENTER THE NUMBER OF REPETITIONS

Section 4.4 - Logical Memory: Brave Man Recall

READ: Now I will tell a short story, then I will ask you to repeat as much of the story as you can remember after I finish reading. I want you to listen very carefully because I want you to try to tell me the whole story with as many details as you can remember.

“Three children were alone at home and the house caught on fire. A brave man managed to climb in a back window and carry them to safety. Aside from minor cuts and bruises, all were well. Now I would like you tell me the story in as much detail as possible”

[***ENUMERATOR NOTE:*** AN APPROXIMATE ANSWER FOR “3 CHILDREN” COULD INCLUDE “SOME CHILDREN” OR “3 PEOPLE” OR “4 CHILDREN.” IF THEY SAY “4 MEN” THAT IS NOT CORRECT.]

Story Recall	0=Not correct, not mentioned | 1=Approximate answer | 2=Exact Answer
3 children	
alone at home	
house on fire	
brave man	
climbed	
back window	

carry them to safety	
minor cuts	
bruises	
all were well	

LM_0001 {IMMEDIATE RECALL BRAVE MAN STORY SCORE= Record the number of exact story points the respondent was able to recall when retelling a story immediately after it was read aloud to him/her. Scores range from 0-6.}

Section 4.5 - Clock Drawing

READ: Now I will ask you to draw a clock for me using this pencil and paper. Please start with a circle. Then draw the numbers on the face of the clock. Finally, draw the hands to show 10 minutes after 11. Please ensure to draw the clock using the English time as it would appear in the real clock.

*[**ENUMERATOR NOTE**: FIRST READ THE INSTRUCTIONS AND THEN HAND THE PAPER AND PEN TO THE RESPONDENT]*

CD_0001 **[DO NOT READ]** IS THERE A CLOSED CIRCLE?

1= Yes

0= No

-77= Don’t know

-88= Refused

CD_0002 **[DO NOT READ]** ARE THE NUMBERS CORRECTLY PLACED AND ORDERED INSIDE THE CIRCLE?

1= Yes

0= No

-77= Don’t know

-88= Refused

CD_0003 **[DO NOT READ]** ARE THERE TWO CLOCK HANDS?

1= Yes

0= No

-77= Don’t know

-88= Refused

CD_0004 **[DO NOT READ]** ARE THE TWO CLOCK HANDS CORRECTLY SHOWING 10 MINUTES AFTER 11?

1= Yes

0= No

-77= Don’t know

-88= Refused

Section 4.6 - Making Change

MC_0001 I will now ask you a question about making change from a 1,000 Ksh note. How many 200 Ksh notes will be given for one 1,000 Ksh note?

1= Correct

0= Incorrect

-77 = Don’t know/doesn’t understand

-88 = Refused

*[**ENUMERATOR NOTE:** THE CORRECT ANSWER IS 5]*

Section 4.7 - 10-Word List Recall: Delayed

READ: Do you remember that I read out to you a list of words? How many of those words do you remember now? Could you please tell me all the words you can remember.

*[**ENUMERATOR NOTE:** MARK THE ORDER IN WHICH THE WORD IS RECALLED. RECORD ALL WORDS THE RESPONDENT SAYS THAT ARE NOT IN THE LIST.]*

**WORDS**	**ORDER**

**MILK**	
**ARM**	
**LETTER**	
**QUEEN**	
**TICKET**	
**GRASS**	
**CORNER**	
**STONE**	
**BOOK**	
**STICK**	
**Other words**	
**TOTAL SCORE**	

CE_delayed_0001 Total number of words correctly recalled

SECTION 5: PSYCHOSOCIAL, MENTAL HEALTH, & BEHAVIORAL

Section 5.1 - Depressive Symptoms

CES-D

READ: I will read out a list of some of the ways you may feel or behave. Please indicate how often you have felt this way during the **past week**:

[ENUMERATOR NOTE: SHOW THE RESPONDENT THE SCALE. DEMONSTRATE THAT THEY SHOULD SELECT THEIR RESPONSE USING THE SCALE. FOR THE REST OF THE QUESTIONS IN THIS SECTION, READ THE QUESTIONS EXACTLY AS WRITTEN. YOU MAY REPEAT ANY QUESTIONS AS MANY TIMES AS YOU'D LIKE, BUT DO NOT REPHRASE ANY QUESTION OR ADD ADDITIONAL COMMENTS OR EXPLANATIONS. IF THE SP HAS TROUBLE UNDERSTANDING THE STATEMENT, PLEASE RE-READ BUT DO NOT TRY TO EXPLAIN THE QUESTION IN A DIFFERENT MANNER]

DP_0001	In the past week, I was bothered by things that usually don't bother me	1= Rarely or none of the time<br><br>2= Some or a little of the time<br><br>3= Occasionally or a moderate amount of time<br><br>4= All of the time<br><br>66= Refuses to respond<br><br>99= Don't know/Doesn't understand
DP_0002	In the past week, I had a problem concentrating on what I was doing	
DP_0003	In the past week, I felt depressed and troubled in my mind	
DP_0004	In the past week, I felt that everything that I did took up all my energy	
DP_0005	In the past week, I felt hopeful about the future	

DP_0006	In the past week, I felt afraid	
DP_0007	In the past week, I had difficulty in sleeping peacefully	
DP_0008	In the past week, I was happy	
DP_0009	In the past week, I felt lonely	
DP_0010	In the past week, I lacked the motivation to do anything	

Section 5.2 - Loneliness

3-item loneliness scale

READ: Here is a list of statements on loneliness. We would like to know how often, if at all, you feel this way

[ENUMERATOR NOTE: SHOW THE RESPONDENT THE SCALE. DEMONSTRATE THAT THEY SHOULD SELECT THEIR RESPONSE USING THE SCALE. FOR THE REST OF THE QUESTIONS IN THIS SECTION, READ THE QUESTIONS EXACTLY AS WRITTEN. YOU MAY REPEAT ANY QUESTIONS AS MANY TIMES AS YOU'D LIKE, BUT DO NOT REPHRASE ANY QUESTION OR ADD ADDITIONAL COMMENTS OR EXPLANATIONS. IF THE SP HAS TROUBLE UNDERSTANDING THE STATEMENT, PLEASE RE-READ BUT DO NOT TRY TO EXPLAIN THE QUESTION IN A DIFFERENT MANNER]

LO_0001	How often do you feel you lack companionship	1=Often<br>2= Some of the time<br>3= Hardly ever or never
LO_0002	How often do you feel left out	
LO_0003	How often do you feel isolated from others	

Section 5.3 - Subjective well-being

CASP- 19

READ: Here is a list of statements that people have used to describe their lives or how they feel. We would like to know how often, if at all, you feel this way

[ENUMERATOR NOTE: SHOW THE RESPONDENT THE SCALE. DEMONSTRATE THAT THEY SHOULD SELECT THEIR RESPONSE USING THE SCALE. FOR THE REST OF THE QUESTIONS IN THIS SECTION, READ THE QUESTIONS EXACTLY AS WRITTEN. YOU MAY REPEAT ANY QUESTIONS AS MANY TIMES AS YOU'D LIKE, BUT DO NOT REPHRASE ANY QUESTION OR ADD ADDITIONAL COMMENTS OR EXPLANATIONS. IF THE SP HAS TROUBLE UNDERSTANDING THE STATEMENT, PLEASE RE-READ BUT DO NOT TRY TO EXPLAIN THE QUESTION IN A DIFFERENT MANNER]

WB_0001	My age prevents me from doing the things I would like to.	1= Often<br>2= Sometimes<br>3= Not Often<br>4= Never
WB_0002	I feel that what happens to me is out of my control.	
WB_0003	I feel free to plan for the future.	

WB_0004	I feel left out of current activities/happenings	
WB_0005	I can do activities that I want to do.	
WB_0006	Family responsibilities prevent me from doing what I want to do.	
WB_0007	I feel that I can do activities as I please	
WB_0008	My health stops me from doing things I want to do	
WB_0009	Shortage of money stops me from doing the things I want to do	
WB_0010	I look forward to each day	
WB_0011	I feel that my life has value/purpose	
WB_0012	I enjoy the activities that I do	
WB_0013	I enjoy being in the company of others.	
WB_0014	On balance, I look back on my life with a sense of happiness	

WB_0015	I feel full of energy these days	
WB_0016	I choose to do activities that I have never done before	
WB_0017	I feel satisfied with the way my life has turned out	
WB_0018	I feel that life is full of opportunities	
WB_0019	I feel that the future looks good for me	

Section 5.4 - Ill Treatment

[ENUMERATOR NOTE: MAKE SURE SP IS ALONE IN THE ROOM WHEN ANSWERING THIS SECTION]

IT_0001. Have you felt that you were ill-treated in the past year?

a. Yes

b. No **Skip to Section 5.5**

IT_0002. [Ask only if IT_0001=a] How often did you feel that way?

a. Frequently

b. Occasionally

c. Only few times

IT_0003. Who are the persons who ill-treated you during the last one year? [Instructions for CAPI: Multiple answers are allowed]

a. Spouse/partner

b. Son/s

c. Daughter/s

d. Son-in-law

e. Daughter-in-law

f. Grandchildren

g. Brother

h. Sister

i. Other Relatives

j. Neighbors

k. Husband’s brother/s

l. Other, please specify

IT_0004. What kind of ill-treatment did you face during the last one year?

[ENUMERATOR NOTE: FOR EACH TYPE, ASK IF WITHIN HH OR OUTSIDE HH]

Sr. no	Type of ill-treatment	Within Household	Outside Household
a.	Physical	1. Yes<br>2. No	1. Yes<br>2. No
b.	Verbal/Disrespect	1. Yes<br>2. No	1. Yes<br>2. No
c.	Economic exploitation	1. Yes<br>2.No	1. Yes<br>2. No

d.	Emotional/Psychological	1. Yes<br>2. No	1. Yes<br>2. No
e.	Neglect	1. Yes<br>2. No	1. Yes<br>2. No

Section 5.5 - Life Satisfaction

Single Item Life Satisfaction:

LS_0001 Please think about your life-as-a-whole. How satisfied are you with it? Are you completely satisfied, very satisfied, somewhat satisfied, not very satisfied, or not at all satisfied?

1. COMPLETELY SATISFIED
2. VERY SATISFIED
3. SOMEWHAT SATISFIED
4. NOT VERY SATISFIED
5. NOT AT ALL SATISFIED
8. DK (Don't Know), NA (Not Ascertained)
9. RF (Refused);

Diener's Life satisfaction scale:

LS_0002 Please say if you strongly agree, somewhat agree, slightly agree, neither agree nor disagree, slightly disagree, somewhat disagree or strongly disagree with the following statements.

	Strongly disagree	Somewhat disagree	Slightly disagree	Neither agree nor disagree	Slightly Agree	Somewhat Agree	Strongly Agree
a. In most ways my life is close to ideal.	1	2	3	4	5	6	7
b. The conditions of my life are excellent.	1	2	3	4	5	6	7
c. I am satisfied with my life.	1	2	3	4	5	6	7
d. So far, I have got the important things I want in life.	1	2	3	4	5	6	7

e. If I could live my life again, I would change almost nothing.	1	2	3	4	5	6	7

Section 5.6 - MacArthur Ladder

*[**ENUMERATOR NOTE**: SHOW THE RESPONDENT THE SCALE WITH THE PICTURE OF A LADDER. DEMONSTRATE THAT THEY SHOULD SELECT THEIR RESPONSE USING THE DIAGRAM]*

[Instructions for CAPI: make sure ladder fills entire tablet screen.]

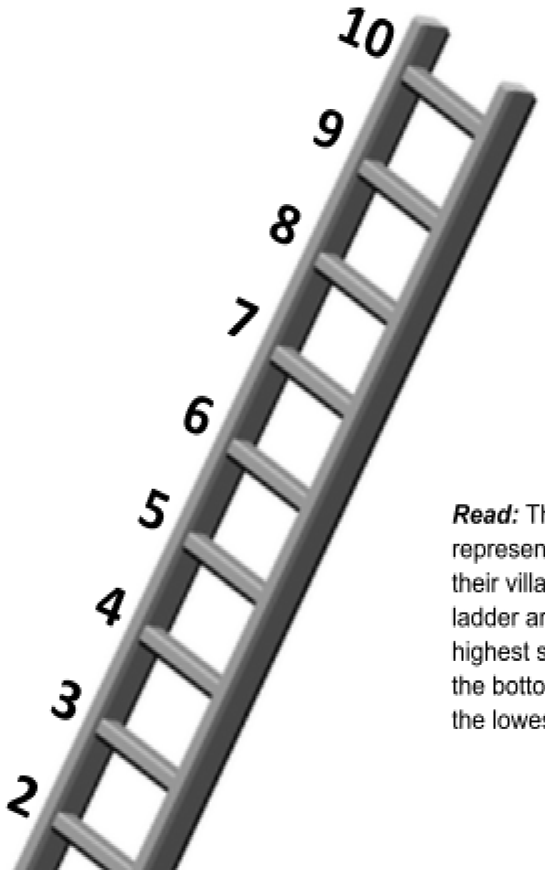

Read: Think of a ladder as representing where people stand in their villages. At the top of the ladder are the people who have the highest standing in their village. At the bottom are the people who have the lowest standing in their village.

ML_0001 Where would you place yourself on the ladder?
1. 1-10

8. DK (Don't Know), NA (Not Ascertained)

9. RF (Refused);

ML_0002 What place on the ladder would you like to achieve in your life?
1. 1-10

8. DK (Don't Know), NA (Not Ascertained)

9. RF (Refused);

Section 5.7 - 4-item Perceived Stress Scale

READ: The questions in this scale ask you about your feelings and thoughts during THE LAST MONTH. In each case, please indicate your response under following scale:

PSS_0001	In the last month, how often have you felt that you were unable to control the important things in your life?	0- Never<br>1- Almost never<br>2- Sometimes<br>3- Fairly often<br>4- Very often
PSS_0002	In the last month, how often have you felt confident about your ability to handle your personal problems?	4 = Never<br>3 = Almost Never<br>2 = Sometimes<br>1 = Fairly Often<br>0 = Very Often

PSS_0003	In the last month, how often have you felt that things were going your way?	4 = Never<br>3 = Almost Never<br>2 = Sometimes<br>1 = Fairly Often<br>0 = Very Often
PSS_0004	In the last month, how often have you felt difficulties were piling up so high that you could not overcome them?	0- Never<br>1- Almost never<br>2- Sometimes<br>3- Fairly often<br>4- Very often

[ENUMERATOR NOTE: NOTE THE RESPONSE OPTIONS ARE NOT SAME FOR ALL 4 QUESTIONS. MARK ACCORDINGLY]

Section 5.8 - Financial strain

FS_0001 How difficult is it for you (and your family) to pay for the very basics like food, medical care, and heating?

1 = very hard

2 = hard

3 = somewhat hard

4 = not very hard.

***NOTE: For the purpose of analysis, financial strain is re-coded: very hard and hard as high strain and somewhat and not very hard as low strain (DO NOT INCORPORATE THIS ON CAPI)**

SECTION 6: OTHER SELF-REPORTED HEALTH MEASURES & RISK FACTORS

***[ENUMERATOR NOTE:** THE QUESTIONS IN THIS SECTION ARE MORE PERSONAL. TRY TO ENSURE PRIVACY OF THE RESPONDENT]*

Section 6.1 - Self-Reported Health Status

SRHS_0001 Would you describe your general health as very good, somewhat good, or not good?

1= Very Good

2= Somewhat good

3= Not good

SRHS_0002 *[**ENUMERATOR NOTE**: ASK ONLY IF SRHS_0001= 2 OR =3]* Would you describe your general health as good, fair, poor, or very poor?

1= Good

2= Fair

3= Poor

4= Very poor

Section 6.2 - Disabilities

Washington Group Short Set on Disability

Dis_0001	Do you have difficulty seeing, even if wearing glasses?	1= No-no difficulty<br>2= Yes-some difficulty<br>3= Yes-a lot of difficulty<br>4=Cannot do at all<br>66=Refuses to answer
Dis_0002	Do you have difficulty hearing, even if using a hearing aid?	
Dis_0003	Do you have difficulty walking or climbing steps?	
Dis_0004	Do you have difficulty remembering or concentrating?	
Dis_0005	Do you have difficulty with self-care such as washing all over or dressing?	

Dis_0006	Using your usual language, do you have difficulty communicating, for example understanding or being understood?	

SECTION 7: ENVIRONMENT

Section 7.1 - Air Pollution

AP_0001 Does this household have electricity?
1. Yes
2. No

AP_0002 In your household, what type of cookstove is mainly used for cooking?
1. Electric stove
2. Solar cooker
3. Liquefied Petroleum Gas (LPG)
4. Piped natural gas stove
5. Biogas stove
6. Manufactured solid fuel stove
7. Traditional solid fuel stove (example: jiko ya makaa)
8. Three stone stove/open fire
9. No food cooked in household
10. Other, please specify___________

AP_0003. [**ENUMERATOR NOTE**: ASK ONLY IF AP_0002 IS NOT 9]

How long have you been using [Fuel] for cooking?

___________________Years

AP_0004. Does the stove have a chimney?

1. Yes
2. No

AP_0005. Does the stove have a fan?

1. Yes
2. No

AP_0006. What type of fuel or energy source is used in this cookstove?

1. Alcohol/Ethanol
2. Gasoline/Diesel
3. Kerosene/Paraffin
4. Coal/Lignite
5. Charcoal
6. Wood
7. Straw/Shrubs/Grass
8. Agricultural crop
9. Animal dung/Waste
10. Processed Biomass (Pellets) or Woodchips
11.Garbage/Plastic
12. Sawdust

13. Other, please specify ____________

AP_0007. For how many hours and minutes in a day does your household use [fuel or energy source] for cooking or boiling water or any other purposes?

[**ENUMERATOR NOTE**: ENTER ‘0’ IN HOURS BOX IF DURATION IS MENTIONED IN MINUTES. ENTER ‘0’ IN MINUTES BOX IF DURATION IS MENTIONED IN HOURS]

_____ Hours AND _____Minutes per day

AP_0008. Is the cooking usually done in the house, in a separate building, or outdoors?

1. In the house
2. In a separate building
3. Outdoors
4. Other, please specify __________

AP_0009. Do you have a separate room which is used as a kitchen?

1. Yes
2. No

AP_0010. What does this household use to light the home when needed? Select all that apply
[Instructions for CAPI: Multiple answers allowed]

1. Electricity
2. Solar Lantern
3. Rechargeable Flashlight, torch or lantern
4. Battery powered flashlight, torch or lantern
5. Biogas lamp
6. Gasoline lamp
7. Kerosene or Paraffin lamp
8. Charcoal

9. Wood

10. Straw/Shrubs/Grass

11. Agricultural crop

12. Animal dung/waste

13. Oil lamp

14. Oil lantern flame

15. Candle

16. No lighting in household

17. Other, please specify _____________-

AP_0011. Does any usual member of your household smoke inside the home?

1. Yes

2. No

AP_0011.1 [Ask only if AP_0011=1] How many hours and minutes per day does he/she/they smoke inside the home?

[**ENUMERATOR NOTE**: ENTER ‘0’ IN HOURS BOX IF DURATION IS MENTIONED IN MINUTES. ENTER ‘0’ IN MINUTES BOX IF DURATION IS MENTIONED IN HOURS]

_____ Hours AND _____Minutes per day

Section 7.2 - Climate vulnerability

CV_0001 During the last 12 months, was your household affected negatively by any of the following events? [Instructions for CAPI: Multiple answers allowed]

1. Drought

2. Irregular rains

3. Floods

4. Landslides

5. Earthquakes

6. Unusually high level of crop pests or disease

7. Unusually high level of livestock pests or disease

8. Unusually low prices for agricultural inputs

9. Unusually high costs of agricultural inputs

10. Unusually high prices for food

11. Lower agricultural output than expected

12. Other, please specify __________

CV_0002 Rank the three most significant events you experienced. Select top three from the above list

1. Most severe ________

2. Second most severe ________

3. Third most severe _________

Section 7.3 - Food and Water insecurity

FW_0001 In the past [4 weeks/30 days], was there ever no food of any kind to eat in your house because of lack of resources to get food?

1. Yes

2. No

3. Don't know

FW_0002 In the past [4 weeks/30 days], did you or any household member go to sleep at night hungry because there was not enough food?

1. Yes

2. No

3. Don't know

FW_0003 In the past [4 weeks/30 days], did you or any household member go a whole day and night without eating anything at all because there was not enough food?

1. Yes
2. No
3. Don't know

READ: for the following questions, please indicate your response under the following scale

1. Never (0 times)
2. Rarely (1-2 times)
3. Sometimes (3-10 times)
4. Often (11-20 times)
5. Always (more than 20 times)
6. Do not know
7. Not applicable/I do not have this

FW_0004 In the last 4 weeks, how frequently did you or anyone in your household worry you would not have enough water for all of your household needs?

FW_0005 In the last 4 weeks, how frequently have you or anyone in your household had to change schedules or plans because of problems with your water situation? (Activities that may have been interrupted include caring for others, doing household chores, agricultural work, income-generating activities, and sleeping)

FW_0006 In the last 4 weeks, how frequently has there not been as much water to drink as you would like for you or anyone in your household?

FW_0007 In the last 4 weeks, how frequently have you or anyone in your household had to go without washing hands after dirty activities (e.g., defecating, changing diapers, cleaning animal dung) because of problems with water?

SECTION 8: ECONOMICS

Section 8.1 : Household income

HI_0001. Are you currently working?
1. Yes

2. No

HI_0002. What was/is your primary occupation?
1. FARMING/ANIMAL HUSBANDRY

2. RETAIL/PETTY SHOP

3. WHOLESALE DEALER

4. MILL OWNER

5. AGRI INPUT SHOP PESTICIDES, ETC

6. CYCLE/AUTO REPAIR SHOP

7. FRUIT VENDOR

8. VEGETABLE VENDOR

9. CHICKEN/MEAT SHOP

10. FISH SELLER

11. CAMERA/VIDEO/STUDIO WORK

12. CELL PHONE SERVICE

13. SOUNDS SERVICE

14. LAUNDRY/IRONING SERVICE

15. CARPENTRY

16. BARBER

17. TAILOR SHOP

18. CAR RENTAL

19. MILK VENDING

20. MONEY LENDING

21. FARM LABORER

22. NON- FARM (UNSKILLED)

23. PRIVATE FORMAL SALARY JOB

24. GOVERNMENT JOB

25. ELECTRICIAN

26. DRIVER

27. WOODWORKER

28. TRADITIONAL SILK WEAVING INDUSTRY

29. BODABODA OR TUK-TUK OPERATOR

30. REFUSED TO ANSWER

31. OTHERS, SPECIFY

-88 DO NOT KNOW

HI_0003**.** What was the total number of days you worked in this type of work in the past 7 days?
*[**ENUMERATOR NOTE**: ASK ONLY IF HI_0001 = 1]*

1. ONE DAY

2. TWO DAYS

3. THREE DAYS

4. FOUR DAYS

5. FIVE DAYS

6. SIX DAYS

7. SEVEN DAYS

8. N/A

HI_0004. In the past 3 months what is the total income you generated from this activity?
(Instructions for CAPI: Name the activity based on response from HI_0002)

*[**ENUMERATOR NOTE**: ASK ONLY IF HI_0001 = 1]* ___________ Ksh

HI_0005. Is your job full-time/part-time/seasonal?
1. Full-time

2. Part-time

3. Seasonal

-88. Do not know

HI_0006. In the last 3 months, did you have any other occupation/employment?
1. Yes

2. No

HI_0006a. If yes, in which sector(s) did you work? (select all that apply)
1. FARMING/ANIMAL HUSBANDRY

2. RETAIL/PETTY SHOP

3. WHOLESALE DEALER

4. MILL OWNER

5. AGRI INPUT SHOP PESTICIDES, ETC

6. CYCLE/AUTO REPAIR SHOP

7. FRUIT VENDOR

8. VEGETABLE VENDOR

9. CHICKEN/MEAT SHOP

10. FISH SELLER

11. CAMERA/VIDEO/STUDIO WORK

12. CELL PHONE SERVICE

13. SOUNDS SERVICE

14. LAUNDRY/IRONING SERVICE

15. CARPENTRY

16. BARBER

17. TAILOR SHOP

18. CAR RENTAL

19. MILK VENDING

20. MONEY LENDING

21. FARM LABORER

22. NON- FARM (UNSKILLED)

23. PRIVATE FORMAL SALARY JOB

24. GOVERNMENT JOB

25. ELECTRICIAN

26. DRIVER

27. WOODWORKER

28. TRADITIONAL SILK WEAVING INDUSTRY

29. BODABODA OR TUK-TUK OPERATOR

30. REFUSED TO ANSWER

31. OTHERS, SPECIFY

-88 DO NOT KNOW

HI_0006b. What was the total number of days you worked in this type of work in the past 7 days?

1. ONE DAY
2. TWO DAYS
3. THREE DAYS
4. FOUR DAYS
5. FIVE DAYS
6. SIX DAYS
7. SEVEN DAYS
8. N/A

HI_0006c. In the past 3 months what is the total income you generated from this activity? (Instructions for CAPI: Name the activity based on response from HI_0006a)

*[**ENUMERATOR NOTE**: ASK ONLY IF HI_0006 = 1]* _______________Ksh

HI_0007 In the last 3 months, what was the approximate total amount earned by all household members from all income-generating activities?

***[ENUMERATOR NOTE:** INCLUDE IN-KIND EARNINGS, BUT FIRST CONVERT TO CASH AND THEN ADD TO THE TOTAL]* ____________ Ksh

Section 8.2 - Retirement

RT_0001. Did you officially retire from your primary occupation

1. Yes
2. No

RT_0002.*[**ENUMERATOR NOTE:** ASK ONLY IF THE RESPONDENT IS CURRENTLY WORKING OR TEMPORARILY NOT WORKING. SKIP IF RT_0001=1]*

At what age do you plan to stop working? Stopping work in this context shall refer to having stopped all income-related activities on a regular basis and having no intention of engaging in any income related activities seriously.

Please tell me the approximate age when you plan to stop work. *[**ENUMERATOR NOTE**: ENTER ZERO '0' IF SP PLANS TO RETIRE IN LESS THAN A YEAR]*

1. Years old ______ **[Soft check < 55]** OR

2. Years in the future ________ OR

3. SP plans to keep working as long as he/she is physically capable (voluntary)

RT_0003. [***ENUMERATOR NOTE**: ASK ONLY IF RT_0001 = 1*] In which month and year did you take official retirement from your last job?

Year _______

Month ______

RT_0004. [***ENUMERATOR NOTE**: ASK ONLY IF RT_0001 = 1*] What is the mandatory retirement age at the work unit you retired from in the last job?

Age _____

RT_0005. [***ENUMERATOR NOTE**: ASK ONLY IF RT_0001 = 1*] Was your official retirement: early retirement, at the mandatory retirement age, or later than the mandatory retirement age?

1. Early retirement

2. Mandatory retirement age

3. Later than the mandatory retirement age

RT_0006 [***ENUMERATOR NOTE**: ASK ONLY IF RT_0005 = 1*] What was the **main** reason you chose to retire early?

1. Got better job opportunity

2. Already had enough income to get by

3. Had enough income from spouse

4. Didn’t want to continue to work

5. To spend more time on leisure

6. To do volunteer work or to pursue hobbies

7. My job was classified as high-risk or hard manual labor, and therefore I was eligible for early retirement

8. I completed the minimum number of years required for obtaining pension benefits

9. My work unit was restructuring/bankrupt, so I was offered early retirement

10. Due to poor health of a spouse or another family member

11. Due to my own poor health

12. Due to child rearing or housekeeping

13. Other, please specify ___________

RT_0007 [***ENUMERATOR NOTE***: *ASK ONLY IF RT_0001 = 1*] What is your best estimate of your pre-retirement salary (last drawn monthly pay) at the work unit from which you officially retired? ___________Ksh in last month

Section 8.3 - Cash Transfers

CT_0001.Is any member of your household (HH) part of a social welfare program from Kenyan Government?

1. Yes
2. No

CT_0002 [***ENUMERATOR NOTE***: *ASK ONLY IF CT_0001 = 1*] Is the HH member part of any of the following programs: [Instructions for CAPI: Multiple answers allowed]

1. Monthly Pension payment (60+)

2. Dependants’ pension

3. Mother and Child Cash Transfer (MCCT)

4. One time cash transfer (80-84) social pension (CERP[1])

5. One time cash transfer (MCCT – Ngwe Bike) (as part of CERP)

6. Relief food Support (as part of CERP)

7. Cash transfer for elderly persons (70+ years)

8. Cash transfer for person with disability

9. COVID-19 Economic Relief Plan

CT_0003. If part of social program, kindly provide the following details: [CAPI Instructions: These questions need to be answered for each social program they are part of]

CT_0003a. How long have you received this?________ years

CT_0003b. How often do you receive it?
1. Monthly

2. Quarterly (every three months)

3. Don't have the regular interval

4. Don't Know

5. Other, please specify ___

CT_0003c. What amount do you get during each visit? ___Ksh

CT_0003d. Did you ever NOT receive the amount owed?
1. Yes

2. No

CT_0003e. If not, why not? List all relevant problems __________

CT_0003f. Do you go to collect this or send somebody else?
1. Self

2. Somebody else - specify _______

CT_0003g. If you're going to collect it, how do you reach the collection center?

CT_0003h. What do you use the cash for?
1. Buy groceries

2. Pay for house-related items

3. Pay for personal expenses

4. Give to other family members – voluntarily

5. Give to other family members - involuntarily

Section 8.4 - Household assets module

HA_0001 What type of fuel does your household MAINLY use for cooking?

[**CAPI Instructions**: Refer to Section 7.1, Question AP_0006, answer from it must be auto-populated here]

HA_0002 How many rooms in this household can be used for sleeping

_________ rooms

HA_0003 Does this household own any livestock, herds, other farm animals, or poultry?
1. Yes
2. No
3. Don’t know

HA_0004 How many of the following animals does this household own?
1. Local cattle
2. Exotic/grade cattle
3. Horses/donkeys/camels
4. Goats
5. Sheep
6. Chicken
7. None

HA_0005 Does any member of the household own agricultural land?
1. Yes
2. No
3. Don’t know

HA_0006 How many acres of agricultural land do members of this household own?

__________ acres

HA_0007 Does your household have: (1 = Yes, 2 = No)
1. Electricity
2. A radio
3. A television
4. A refrigerator/freezer
5. Solar panel
6. Table
7. Chair
8. Bed
9. Cupboard
10. Clock
11. DVD Player
12. Cassette or CD player

HA_0008 Does any member of your household own: (1 = Yes, 2 = No)
1. A watch
2. A bicycle
3. A mobile telephone
4. A motorcycle or scooter
5. A car or truck

HA_0009 Main material of the dwelling floor [***ENUMERATOR NOTE****: RECORD FROM OBSERVATION*]
1. Natural floor

A1. Earth / Sand

A2. Dung

2. Rudimentary floor
B1. Wood planks

B2. Palm/bamboo

3. Finished floor
C1. Parquet or polished wood

C2. Vinyl or asphalt strips

C3. Ceramic tiles

C4. Cement

C5. Carpet

4. Other, please specify ____________

HA_0010 Main material of the roof ***[ENUMERATOR NOTE**: OBSERVE AND MARK THE RESPONSE]*

1. Natural roofing
A1. No roof

A2. Thatch/Grass/Makuti

A3. Dung/Mud/Sod

2. Rudimentary roofing
B1. Iron sheets

B2. Tin cans

3. Finished roofing
C1. Asbestos sheet

C2. Concrete

C3. Tiles

4. Other, please specify ____________

HA_0011 Main material of the external wall ***[ENUMERATOR NOTE**: OBSERVE AND MARK THE RESPONSE]*

1. Natural walls

A1. No walls

A2. Cane/Palm/Trunks

A3. Dung/Mod/Sod

2. Rudimentary walls

B1. bamboo with mud

B2. Stone with mud

B3. Uncovered adobe

B4. Plywood

B5. Cardboard

B6. Reused wood

B7. Iron sheets

3. Finished walls

C1. Cement

C2. Stone with lime/cement

C3. Bricks/stone

C4. Cement blocks

C5. Covered adobe

C6. Wood planks/shingles

4. Other, please specify ____________

SECTION 9: CAREGIVING EXPOSURE AND STRESS

CG_0001 Are you currently providing care on an on-going basis to a family member, friend, or neighbor with a chronic illness or a disability? This would include any kind of regular help with basic activities such as dressing, bathing, grooming this person, managing bills, arranging for medical care, watching, or supervising this person, or providing transportation?

0=No

1=Yes

CG_0002 [**ENUMERATOR NOTE**: ASK ONLY IF CG_0001=1]What is his or her relationship to you?

01 Mother

02 Father

03 Mother-in-law

04 Father-in-law

05 Child

06 Husband

07 Wife

08 Live-in partner

09 Brother or brother-in-law

10 Sister or sister-in-law

11 Grandmother

12 Grandfather

13 Grandchild

14 Other relative

15 Non-relative/ Family friend

77 Don’t know/Not sure

99 Refused

CG_0003 [**ENUMERATOR NOTE**: ASK ONLY IF CG_0001=1]] Are you the primary person responsible for providing care to this family member, friend, or neighbor with a chronic illness or disability?

1= I am the primary caretaker

2= Someone else in the home is the primary person caring for their needs.

3= I share the responsibility with other family members.

4= They care primarily for themselves.

CG_0004 [**ENUMERATOR NOTE**: ASK ONLY IF CG_0001=1] How many people are you caring for on a daily basis?

____ number

CG_0005 [**ENUMERATOR NOTE**: ASK ONLY IF CG_0001=1] In a typical day, how many hours do you spend caregiving?

_____ hours/day (soft check <24 hours)

Positive affect

CG_0006 Thinking about the last month, how often did you feel cheerful?

1= every day

2= most days

3= some days

4= rarely

5= never

CG_0007 Thinking about the last month, how often did you feel calm and peaceful?
1= every day

2= most days

3= some days

4= rarely

5= never

CG_0008 [**ENUMERATOR NOTE**: ASK ONLY IF CG_0001=1] How much do you agree with the following statement: Completing duties within my home and caring for members of my household is deeply meaningful to me.
1 = strongly disagree

2 = disagree

3 = neither agree nor disagree

4 = agree

5 = strongly agree

6 = not applicable

READ: I am going to read a list of things that other people have found to be difficult and tell me if it applies to you or not, and if so, how often?

CG_0009 My sleep is disturbed (For example: the person I care for is in and out of bed or wanders around at night)
1 = Yes, on a regular basis

2 = Yes, sometimes/not very often

5 = No

CG_0010 Caregiving is inconvenient (For example: helping takes so much time or it’s a long walk/commute to go over to help)

1 = Yes, on a regular basis

2 = Yes, sometimes

5 = No

CG_0011 Caregiving is a physical strain (For example: lifting the person in or out of a chair; effort or concentration is required when caregiving)

1 Yes, on a regular basis

2 Yes, sometimes

5 No

CG_0012 Caregiving is confining (For example: helping restricts free time or I cannot go visiting)

1 Yes, on a regular basis

2 Yes, sometimes

5 No

CG_0013 There have been family adjustments (For example: helping has disrupted my routine; there is no privacy)

1 Yes, on a regular basis

2 Yes, sometimes

5 No

CG_0014 There have been changes in personal plans (For example: I had to turn down a job or work)

1 Yes, on a regular basis

2 Yes, sometimes

5 No

CG_0015 There have been other demands on my time (For example: other family members need me)

1 Yes, on a regular basis

2 Yes, sometimes

5 No

CG_0016 There have been emotional adjustments (For example: severe arguments about caregiving with family or with cared for person)

1 Yes, on a regular basis

2 Yes, sometimes

5 No

CG_0017 Some behavior of the cared for person is upsetting (For example: incontinence; the person cared for has trouble remembering things: or the person I care for accuses people of taking things)

1 Yes, on a regular basis

2 Yes, sometimes

5 No

CG_0018 It is upsetting to find the person I care for has changed so much from his/her former self (For example: he/she is a different person than he/she used to be)

1 Yes, on a regular basis

2 Yes, sometimes

5 No

CG_0019 There have been work adjustments (For example: I must take time off work for caregiving duties)

1 Yes, on a regular basis

2 Yes, sometimes

5 No

CG_0020 Caregiving is a financial strain

1 Yes, on a regular basis

2 Yes, sometimes

5 No

CG_0021 I feel completely overwhelmed (For example: I worry about the person I care for; I have concerns about how I will manage)

1 Yes, on a regular basis

2 Yes, sometimes

5 No

SECTION 10: PHYSIOLOGICAL AND ANTHROPOMETRIC MEASURES

Section 10.1 - Blood pressure

[Equipment needed: Omron HEM-780N Monitor, Batteries, Stopwatch]

I would like to measure your blood pressure and pulse using this monitor and cuff which I will secure around your left arm. I would like to take three blood pressure measures. I will ask you to relax and remain seated and quiet, with legs uncrossed and feet flat on the floor, during the measurements. First, I will place the cuff on your left arm. Once the cuff is placed appropriately on your arm and we are ready to begin, I will ask you to lay your arm on a flat surface, palm facing up, so that the center of your upper arm is at the same height as your heart. I will then press the start button. The cuff will inflate and deflate automatically. It will squeeze your arm a bit, but won't hurt. After we have completed all three measures, I will give you your results.

BP_0001. Do you understand these directions and are you willing to provide this measurement?

1. Yes
2. No

BP_0002. Did you smoke, exercise, or consume alcohol or food within the 30 minutes prior the blood pressure test?

1. Yes
2. No

BP_0003. Do you have a rash, a cast, edema (swelling) in the **left arm,** open sores or wounds, or a significant bruise where the blood pressure cuff will be in contact?

1. Yes
2. No

BP_0004. [Ask only if BM003=1]Do you have a rash, a cast, edema (swelling) in the **right arm**, open sores or wounds, or a significant bruise where the blood pressure cuff will be in contact?

1. Yes

2. No

BP_0005. *[**ENUMERATOR NOTE**: WHEN THE DEVICE IS IN THE CORRECT POSITION AND THE SP IS RELAXED, PRESS THE START BUTTON. MEASURE BLOOD PRESSURE AND PULSE THREE TIMES WITH ONE MINUTE GAP BETWEEN EACH OF THE MEASUREMENTS. NO NEED TO REMOVE THE CUFFS AND THE DEVICE BETWEEN THE MEASUREMENTS. ENTER 993 IN FIRST SYSTOLIC READING IF SP TRIED BUT WAS UNABLE TO DO IT/IF AN UNRESOLVABLE EQUIPMENT PROBLEM OCCURS. ENTER 999 IF SP CHOSE NOT TO DO IT. IF THE LOWEST READING OBTAINED IS GREATER THAN 140 SYSTOLIC OR GREATER THAN 90 DIASTOLIC, MAKE A NOTE IN CAPI]*

[Instructions for CAPI: Leave a blank space for any notes at end of module]

Measurement#	Time of reading	Systolic Reading	Diastolic Reading	Pulse
1	am/pm	mmHg	mmHg	beats/min
2	am/pm	mmHg	mmHg	beats/min
3	am/pm	mmHg	mmHg	beats/min
4	Average of last 2 readings*	mmHg	mmHg	beats/min

[*Instructions for CAPI: Average should be auto-populated]

BP_0006. Which arm was used to conduct the measurements?

1. Left arm

2. Right arm

BP_0007. What was SP’s position for this test?

1. Standing

2. Sitting

3. Lying down

BP_0008. How compliant was SP during this measurement?
1. Was fully compliant

2. Was prevented from fully complying due to illness, pain, or other symptoms or discomfort

3. Was not fully compliant

Section 10.2 - Grip Strength/Hand Strength

[Equipment needed: Dynamometer, Stopwatch]

READ: Now I would like to assess the strength of your hand in a gripping action. I will ask you to squeeze this handle as hard as you can, just for a couple of seconds and then let go. I will take alternately two measurements from your right and your left hand. Begin the test with the left hand.

GS_0001. Before we begin, I would like to make sure it is safe for you to do this measurement. Have you had surgery or experienced any swelling, inflammation, severe pain, or injury in one or both hands within the last 6 months?
1. Yes

2. No

GS_0002. [**ENUMERATOR NOTE**: ASK ONLY IF GS_0001=1] In which hand (have you had surgery or experienced any swelling, inflammation, severe pain, or injury in the last 6 months)?

1. Both hands - *[**ENUMERATOR NOTE**: DO NOT COMPLETE THIS MEASURE]*

2. Left hand only - *[**ENUMERATOR NOTE**: CONTINUE BUT DO NOT PERFORM MEASUREMENT ON LEFT HAND]*

3. Right hand only - *[**ENUMERATOR NOTE**: CONTINUE BUT DO NOT PERFORM MEASUREMENT ON LEFT HAND]*

GS_0003.Which is your dominant hand?
1. Right hand

2. Left hand

3. Both hands equally dominant

GS_0004 *[**ENUMERATOR NOTE:** CONDUCT ONE PRACTICE WITH SP'S RIGHT OR LEFT HAND]* **READ:** We can practice with your [right/left] hand.

Start measurement from the Left hand first. Take 30 second-rests between two measurements. Record measurements to the nearest 0.5 kilogram in the table below.

Measurement #	Left hand	Right Hand
1st	_______._ kg	_______._ kg
2nd	_______._ kg	_______._ kg

GS_0005. How much effort did SP give to this test?

1. SP gave full effort

2. SP was prevented from giving full effort by illness, pain, or other symptoms or discomforts

3. SP did not appear to give full effort, but no obvious reason for this

GS_0006 What was SP's position for this test?

1. Standing

2. Sitting

3. Lying down

GS_0007. Did SP rest their arm on a support while performing the test?

1. Yes

2. No

Section 10.3 - Height

[Equipment needed: Stadiometer]

HG_0001 Can the respondent stand?

a. Yes

b. No *[**ENUMERATOR NOTE**: SKIP THIS SECTION]*

HG_0002 Next, I would like to measure your height. To complete this measurement, I will be asking you to take off your shoes and stand up against a wall. Please stand straight and sturdy, and keep step onto the base of the stadiometer, feet together, knees straight, look straight ahead, chin tucked to chest slightly, and do not look up.

*[**ENUMERATOR NOTE**: DEMONSTRATE THE MEASUREMENT. MEASURE IN CENTIMETERS (ROUND OFF TO THE NEAREST 0.1CM)]*

Measurement #	Measurement
1st	____ cm

HG_0003. Was SP wearing any artificial limbs or orthosis during the measurement?

1. Yes

2. No

HG_0004. How compliant was SP during this measurement?

1. Was fully compliant

2. Was prevented from fully complying due to illness, pain, or other symptoms or discomforts

3. Was not fully compliant, but no obvious reason for this

Section 10.4 - Weight

[Equipment needed: Weighing Scale]

WG_0001 Next, I would like to measure your weight. To complete this measurement, I will be asking you to remove bulky clothing and to take off your shoes during weight measurements. Stand up and look straight ahead.

*[**ENUMERATOR NOTE**: DEMONSTRATE THE MEASUREMENT. MEASURE IN KILOGRAMS. ENTER 993 IF SP TRIED BUT RECEIVED AN ERROR]*

Measurement #	Measurement
1st	____ kilograms

WG_0002. Was SP wearing an artificial limb or orthosis during the measurement?

1. Yes, then record the weight of the artificial limb _______

2. No

WG_0003. How compliant was SP during this measurement?

1. Was fully compliant

2. Was prevented from fully complying due to illness, pain, or other symptoms or discomforts

3. Was not fully compliant, but no obvious reason for this

Section 10.5 - Waist Circumference

[Equipment needed: Soft tape measure]

WC_0001 Next I am going to ask you to perform a simple measurement of your waist circumference. For this measurement it is important for you to be standing. I will ask you to identify where on your body your navel (belly button) is located. I will then ask you to place this soft measuring tape around your waist, over your clothing, holding it securely at the level of your navel. Once the tape measure is placed appropriately around your waist then we are ready to

begin. I will ask you to take a normal breath and exhale, holding your breath at the end of the exhale. I will then record the measurement.

*[**ENUMERATOR NOTE:** ENTER '999' IF SP REFUSED. RECORD IN CENTIMETERS (ROUND OFF TO THE NEAREST 0.1 CM]*

Measurement #	Waist Measurement
1st	____ cm

WC_0002. Was SP wearing bulky clothing during this measurement?

1. Yes
2. No

Section 10.6 - Hip circumference

[Equipment needed: Soft tape measure]

HC_0001. Next I am going to ask you to perform a simple measurement of your hip circumference. For this measurement it is important for you to be standing. I will ask you to identify the maximum circumference of your hip. I will then ask you to place this soft measuring tape around your hip, over your clothing, holding it securely. Once the tape measure is placed appropriately around your hip and parallel to floor then we are ready to begin. I will ask you to take a normal breath and exhale, holding your breath at the end of the exhale. I will then record the measurement.

*[**ENUMERATOR NOTE:** ENTER '999' IF SP REFUSED. RECORD IN CENTIMETERS (ROUND OFF TO THE NEAREST 0.1 CM]*

Measurement #	Waist Measurement
1st	____ cm

HC_0002. What difficulties occurred during this measurement? [Instructions for CAPI: Multiple answers are allowed. If HC_0002=1, then freeze other options]

1. None
2. SP had breathing difficulties
3. SP was unable to hold breath at the end of the exhale
4. SP was prevented from giving full effort by illness, pain, or other symptoms or discomforts
5. SP did not appear to give full effort, but no obvious reason for this
6. Had difficulty or unable to locate navel
7. Other, please specify ____________

HC_0003. Who conducted this measurement?

1. SP conducted the measurement
2. Enumerator conducted the measurement

SECTION 11: BIOMARKER COLLECTION

Section 11.1 - Blood sample collection for DBS

I would like to collect a small sample of your blood using just a finger prick. Thank you for agreeing to provide us with a sample of blood. This is an important part of the study, as the analysis of blood samples will tell us a lot about the health of the population.

[Equipment needed: Black spread sheet, DBS Kit, Gloves, Small Biohazard Container, Barcode Scanner and Barcode label, drying rack with Box]

Specimen #	Date	Time
1st	____________ date(dd/mm/yyyy)	_____:_____ am/pm(12hour clock)

BM_0001 ***ENUMERATOR NOTE:***

Step 1. Scan the barcode.

Step 2. Enter the Barcode number twice: BM0001a & BM0001b. [Instructions for CAPI: check BM0001a=BM0001b. If not, re-enter]

BM_0001a Sample Barcode number:…………………………

BM_0001b Sample Barcode number:…………………………

BM_0002.What, if any, problems occurred during the collection of the blood sample?
[Instructions for CAPI: Multiple answers are allowed. If BM_0002=1, then freeze other options]
1. None

2. SP became light-headed, fainted, or nauseous

3. SP had difficulty getting finger to stop bleeding

4. Unable to obtain enough blood

5. Problem with equipment or supplies

6. Other, please specify: ________________________

BM_0003. How many circles were filled on the DBS card?

0 1 2 3 4 5

BM_0004 [For the Field Supervisor]DBS quality assessment by the field supervisor, which is to be completed after they have collected blood spots.

What is the quality of the blood spots that have been collected?

1. Good (will yield five to six 3-mm punches per spot)

2. Fair (will yield three to four 3-mm punches per spot)

3. Poor (will yield one to two 3-mm punches per spot)

4. Spots are unusable

BM_0005.How many times did the SP's finger need to be pricked in order to get 5 drops of blood?

1. One time

2. Twice

3. Twice but the SP was unable to get 5 drops of blood

BM_0006. How compliant was SP during this measurement?

1. Was fully compliant

2. Was prevented from fully complying due to illness, pain, or other symptoms or discomforts

3. Was not fully compliant, but no obvious reason for this

Section 11.2 - Gauging acceptance for saliva sample collection

What is the study about? As part of our ongoing study to understand health and financial situations of aging Kenyans, in the future this research project will collect and analyze genetic data from saliva samples. We hope the genetic data, analyzed in combination with survey responses, will lead to greater understanding of the lives of Kenyans of all ages, including their physical, cognitive and mental health, socio-economic status, happiness, personality, behaviors, choices and their social, family, and work relations and environments.

This genetic data, called DNA, is passed down from your parents to you. It affects your physical characteristics, such as your height, health, and your risk for some diseases. Differences in our genetic code help explain why some people develop certain diseases and others do not, and may play a role in behavior.

From this, we hope to learn, for example, what environments and circumstances lower or increase the genetic risk of developing cognitive impairment and dementia. This knowledge can then be used to identify ways in which the well-being, health and aging of Kenyans can be improved.

Study Procedures: This study will be completely voluntary.

- You will make the decision to participate or not after reviewing information about the study and getting all of your questions answered.
- Participants will provide a saliva sample in a kit that we will send to your home.
- We will extract DNA from the sample, create codes from the DNA, and use those codes as part of our analysis of LOSHAK survey responses.
- The saliva sample will be stored in a facility that meets all standards of the National Institutes of Health and the Kenyan government for data security.
- No identifiers will be associated with the codes we extract to use for analysis.
- Participants can withdraw their consent to participate at any time.

SC_0001 From what you know now, how likely would you be to volunteer to provide a saliva sample for this study? No matter what you tell us now, you will have another chance to make the decision again if the study goes forward.

1. Yes
2. No
3. Not sure

SECTION 12: CONCLUSION

READ: Thank you for your time. Please give me just a moment as I wrap up the survey.

Please make a note if you believe that the information given to you is suspicious:

____

[ENUMERATOR NOTE: *DO NOT READ. SIMPLY RECORD YOUR OWN IMPRESSIONS]*

CON_0000a. Did the SP terminate the survey early?
1=Yes

2=No

If YES, continue. If NO, skip to question CON_0001.

CON_0000b. Why did the SP terminate the survey early?

1 = Temporary stop only – Wishes to continue the survey at a later time. ***See "Temporary Stop Instructions" below.***

2 = Tired

3 = Too busy, does not have time

4 = Offended at question

5 = Suspicious of Field Officer / survey intent / Enumerator

6 = Does not feel like continuing survey

7 = Other, specify

Temporary Stop Instructions: You have indicated that the SP wishes to continue the survey in the future. Please ask the SP when they are next available, and then call your team lead (or other senior team member) to confirm this day and time. If you are unable to confirm this day and time, make a tentative appointment with the SP. Then, let the SP know that you will contact them to confirm when you will return. Record this information and the current time on the tracking sheet now.

CON_0001. Time end interview: (24 hr clock) |__|__| : |__|__|

CON_0002. How was the SP's skill in speaking and understanding Swahili? |____|

1 = Displayed no problems speaking or understanding Swahili

2 = Displayed a little difficulty speaking or understanding Swahili

3 = Displayed moderate difficulty speaking or understanding Swahili

4 = Displayed serious problems speaking or understanding Swahili

CON_0003. Who among the following answered questions in this module? (Indicate all that apply ***1=Yes, 2=No***)

1 = Sample Person (SP) |___|

2 = Sample Person's parents |___|

3 = Sample Person's sibling(s) |___|

4 = Sample Person's spouse(s) |___|

5 = Sample Person's other relatives |___|

6 = Sample Person's other household members who are not relatives |___|

CON_0004. Did the SP become tired or impatient during the survey?

1= Not at all

2=Somewhat tired/impatient

3=Very tired/impatient

CON_0005. How reliable do you think the information in this survey is?

1= Very reliable

2=Somewhat reliable

3= Not at all

CON_0005a. If SOMEWHAT or NOT RELIABLE: Why? ________________________________

FOR NOTES:

____
