# Supplementary material for: Validation of cognitive and psychosocial tools in Kenya: findings from the LOSHAK feasibility pilot
Source: BMC Public Health. 2025 Nov 17;25:4008. doi: 10.1186/s12889-025-24918-z (PMC12625501; doi:10.1186/s12889-025-24918-z)
Supplement: Supplementary file 2 — Additional File 2. Provides summary statistics (mean, standard deviation, minimum and maximum values) for each item in the cognitive and psychosocial domains. It also includes the item threshold plots for each domain. The table of contents is listed below: Supplementary Table S1: Statistics for cognitive domains: Results from LOSHAK (N=203). Supplementary Table S2: Statistics for psychosocial domains: Results from LOSHAK (N=203). Supplementary Figure S1: Person-plot of item thresholds linked to the latent Cognition, Cognitive domains: Results from LOSHAK (N=203). Supplementary Figure S2: Person-plot of item thresholds, Psychosocial domains: Results from LOSHAK (N=203). [file 12889_2025_24918_MOESM2_ESM.pdf]

Supplementary Tables and Figures

Supplementary Table S1: Statistics for cognitive domains: Results from LOSHAK (N=203)

Items	Mean(SD)/ N(%)	Min, Max	Max Value, N (%)
Orientation			
Item 1: Day of month, N (%)	131 (64.9)		
Item 2: Month, N (%)	182 (89.7)		
Item 3: Year, N (%)	103 (51.0)		
Item 4: Day of the week, N (%)	183 (90.1)		
Item 5: What county are we in, N (%)	138 (68.0)		
Item 6: Name of the nearest school?, N (%)	192 (94.6)		
Item 7: Floor, N (%)	196 (96.6)		
Item 8: What city/village are we in, N (%)	193 (95.5)		
Item 9: Season, N (%)	172 (84.7)		
Item 10: Address, N (%)	183 (90)		
Memory			
Item 1: CERAD immediate sum of 3 trials, mean (SD)	13.9 (4.6)	0, 23	1 (0.5%)
Item 2: Brave man immediate (East Boston Memory Test), mean (SD)	4.4 (2.3)	0, 9	6 (3.0%)
Item 3: 3-word delay recall, mean (SD)	2.2 (1.1)	0, 3	115 (56.7%)
Item 4: CERAD word list delay, mean (SD)	3.0 (2.3)	0, 9	1 (0.5%)
Item 5: 3-word immediate recall, mean (SD)	2.9 (0.5)	0, 3	186 (91.6%)
Executive Function			
Item 1: Backward Day naming, N (%)	158 (77.8)		
Item 2: Serial 7s, mean (SD)	2.5 (1.7)	0, 5	25 (14.5%)
Item :3 Making change: How many 200 Ksh in 1000 Ksh note?, N (%)	173 (85.2)		
Item 4: Three-step instruction, mean (SD)	2.6 (0.7)	0, 3	136 (69.0%)
Language			
Item 1: Animal fluency, mean (SD)	10.8 (3.7)	0, 23	1 (0.5%)
Item 2: Watch naming, N (%)	194 (95.6)		
Item 3: Pencil naming, N (%)	198 (97.5)		
Item 4: Repetition of phrase, N (%)	93 (46.0)		

Note: SD: standard deviation.

Supplementary Table S2: Statistics for psychosocial domains: Results from LOSHAK (N=203)

Items	Mean (SD)	Min, Max	Max Value, N (%)
Depressive symptoms (past week): Center for Epidemiological Studies Depression Scale (CES-D)			
Item 1: you were bothered by things that usually don't bother you	1.7 (0.9)	1, 4	7 (3.7%)
Item 2: you had a problem concentrating on what you were doing	1.7 (0.9)	1, 4	5 (2.6%)
Item 3: you felt depressed and troubled in your mind	1.7 (0.9)	1, 4	9 (4.7%)
Item 4: you felt that everything that you did took up all your energy	1.8 (0.9)	1, 4	8 (4.2%)
Item 5: you felt hopeful about the future*	2.4 (1.0)	1, 4	34 (18.2%)
Item 6: you felt afraid	1.7 (0.8)	1, 4	4 (2.1%)
Item 7: you had difficulty sleeping peacefully	1.9 (0.9)	1, 4	10 (5.2%)
Item 8: you were happy*	2.2 (0.9)	1, 4	18 (9.4%)
Item 9: you felt lonely	1.6 (0.9)	1, 4	8 (4.2%)
Item 10you lacked the motivation to do anything	1.8 (0.9)	1, 4	11 (5.8%)
Loneliness: UCLA 3-Item loneliness scale			
Item 1: How often do you feel you lack companionship?	2.5 (0.6)	1, 3	113 (55.7%)
Item 2: How often do you feel left out? For example, in decision-making at home?	2.7 (0.5)	1, 3	136 (67.0%)
Item 3: How often do you feel isolated from others?	2.6 (0.5)	1, 3	120 (59.1%)
Subjective Well-being: Control, Autonomy, Self-Realization and Pleasure (CASP-19) scale			
Item 1: My age prevents me from doing the things I would like to*	2.6 (1.2)	1, 4	53 (26.1%)
Item 2: I feel that what happens to me is out of my control*	2.6 (1.0)	1, 4	35 (17.2%)
Item 3: I feel free to plan for the future	2.0 (1.0)	1, 4	17 (8.4%)
Item 4: I feel left out of current activities/happenings*	2.3 (1.1)	1, 4	29 (14.3%)
Item 5: I can do activities that I want to do	2.3 (1.0)	1, 4	37 (18.2%)
Item 6: Family responsibilities prevent me from doing what I want to do*	2.6 (1.2)	1, 4	60 (29.6%)
Item 7: I feel that I can do activities as I please	2.1 (1.0)	1, 4	26 (12.8%)
Item 8: My health stops me from doing things I want to do*	2.6 (1.1)	1, 4	43 (21.2%)
Item 9: Shortage of money stops me from doing the things I want to do*	3.5 (0.8)	1, 4	134 (66.0%)
Item 10: I look forward to each day	1.8 (0.8)	1, 4	12 (5.9%)
Item 11: I feel that my life has value/purpose	1.6 (0.7)	1, 4	6 (3.0%)
Item 12: I enjoy the activities that I do	1.6 (0.7)	1, 4	5 (2.5%)
Item 13: I enjoy being in the company of others	1.2 (0.5)	1, 4	2 (1.0%)
Item 14: On balance, I look back on my life with a sense of happiness	1.5 (0.7)	1, 4	2 (1.0%)
Item 15: I feel quite vibrant these days	1.9 (0.8)	1, 4	8 (3.9%)
Item 16: I choose to do activities that I have never done before	2.2 (0.9)	1, 4	22 (10.8%)
Item 17: I feel satisfied with the way my life has turned out	2.0 (1.0)	1, 4	20 (9.9%)
Item 18: I feel that life is full of opportunities	1.9 (0.8)	1, 4	12 (5.9%)
Item 19: I feel that the future looks good for me	1.8 (0.9)	1, 4	12 (5.9%)
Life Satisfaction: Satisfaction with Life Scale			
Item 1: In most ways my life is close to ideal	3.6 (2.0)	1, 7	6 (3.0%)
Item 2: The conditions of my life are excellent	3.7 (2.1)	1, 7	14 (6.9%)
Item 3: I am satisfied with my life	3.8 (2.3)	1, 7	13 (6.4%)
Item 4: So far, I have got the important things I want in life	3.7 (2.1)	1, 7	10 (4.9%)
Item 5: If I could live my life again, I would change almost nothing	3.2 (2.2)	1, 7	12 (5.9%)

Notes: *reverse-scored

Supplementary Figure S1: Person-plot of item thresholds linked to the latent Cognition, Cognitive domains: Results from LOSHAK (N=203)

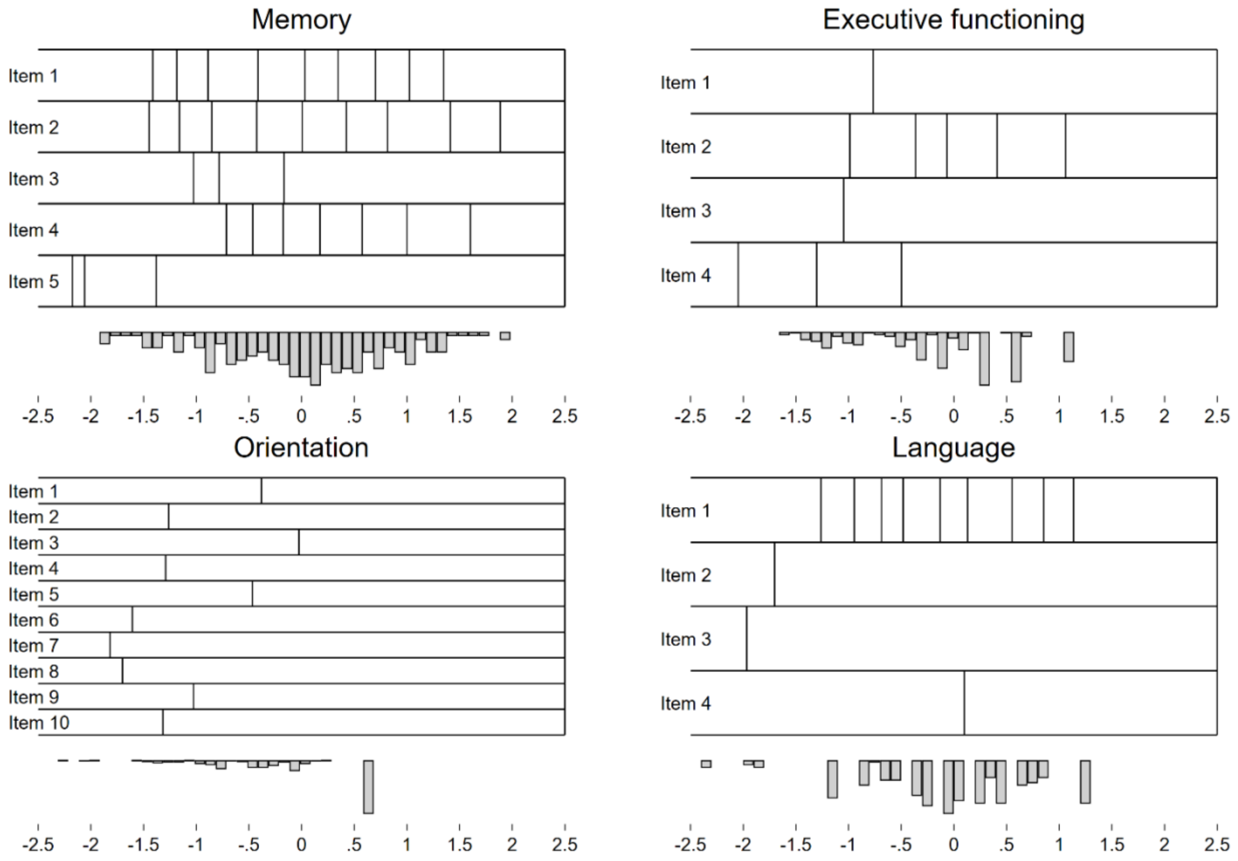

Notes: Refer to Table S1 to map the item numbers in each sub-figure to the corresponding item description within each cognitive domain. For example, "Orientation Item 1" refers to the "Day of the Month."

Supplementary Figure S2: Person-plot of item thresholds, Psychosocial domains: Results from LOSHAK (N=203)

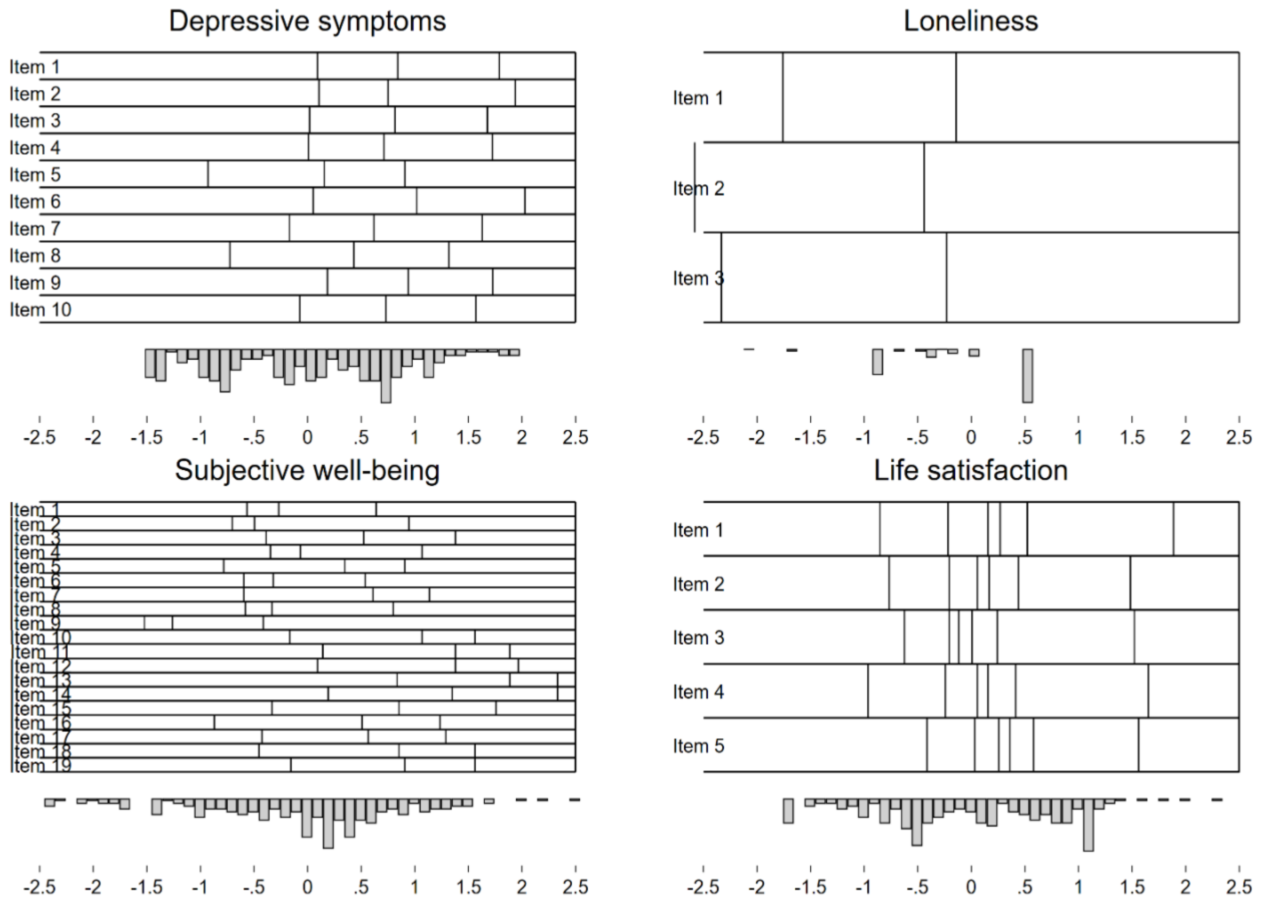

Notes: Refer to Table S2 to match the item numbers with the corresponding item description.
